# A Co-Designed Framework Combining Dome-Aperture Imaging and Generative AI for Defect Detection on Non-Planar Metal Surfaces

**DOI:** 10.3390/s26031044

**Published:** 2026-02-05

**Authors:** Zhongqing Jia, Zhaohui Yu, Chen Guan, Bing Zhao, Xiaofei Wang

**Affiliations:** Shandong Key Laboratory of Optoelectronic Sensing Technologies, National-Local Joint Engineering Laboratory for Energy and Environment Fiber Smart Sensing Technologies, Laser Institute, Qilu University of Technology (Shandong Academy of Sciences), 3501 Daxue Road, Changqing District, Jinan 250353, China; jiazhongqing@vip.sdlaser.cn (Z.J.); 10431230122@stu.qlu.edu.cn (Z.Y.); guanchen1993@qlu.edu.cn (C.G.); zhaobing@sdlaser.cn (B.Z.)

**Keywords:** system co-design, defect imaging, defect image generation, non-planar metallic surface inspection, generative adversarial networks (GANs)

## Abstract

Automated visual inspection of safety-critical metal assemblies such as automotive door lock strikes remains challenging due to their complex three-dimensional geometry, highly reflective surfaces, and scarcity of defect samples. While 3D sensing technologies are often constrained by cost and speed, traditional 2D optical methods struggle with severe imaging artifacts and poor generalization under few-shot conditions. This work constructs a complete system integrating defect imaging, generation, and detection. It proposes an integrated framework through the co-design of an image acquisition system and deep generative models to holistically enhance defect perception capability. First, we develop an imaging system using dome illumination and a small-aperture lens to acquire high-quality images of non-planar metal surfaces. Subsequently, we introduce a dual-stage generation strategy: stage one employs an improved FastGAN with Dynamic Multi-Granularity Fusion Skip-Layer Excitation (DMGF-SLE) and perceptual loss to efficiently generate high-quality local defect patches; stage two utilizes Poisson image editing and an optimized loss function to seamlessly fuse defect patches into specified locations of normal images. This strategy avoids modeling the complete complex background, concentrating computational resources on creating realistic defects. Experiments on a dedicated dataset demonstrate that our method can efficiently generate realistic defect samples under few-shot conditions, achieving 11–24% improvement in Fréchet Inception Distance (FID) scores over baseline models. The generated synthetic data significantly enhances downstream detection performance, increasing YOLOv8’s mAP@50:95 from 50.4% to 60.5%. Beyond proposing individual technical improvements, this research provides a complete, synergistic, and deployable system solution—from physical imaging to algorithmic generation—delivering a computationally efficient and practically viable technical pathway for defect detection in highly reflective, non-planar metal components.

## 1. Introduction

In safety-critical industries such as automotive and aerospace, the structural integrity of metal assemblies (e.g., automotive door lock strikes) forms the cornerstone of product reliability and user safety [1]. Current industrial production primarily relies on two-dimensional vision inspection systems based on optical imaging. However, unlike objects in standard datasets with simple geometries and uniform textures, such as MVTec [2], such industrial components typically possess complex three-dimensional features formed through multiple processes like stamping and riveting, and their surfaces encompass heterogeneous areas such as highlights, rough textures, and transition zones [3]. This complexity makes the imaging results extremely sensitive to lighting angles, surface curvature, and positional variations. Under conventional lighting, optical interferences like specular reflections, local overexposure, and irregular shadows are prone to occur [4], severely obscuring the genuine features of micro defects such as cracks and scratches, resulting in a high miss rate. Concurrently, the exceptionally high production yield in industry leads to extremely scarce defect samples, making it difficult for supervised learning methods that rely on large amounts of annotated data to be directly applied [5,6].

While 3D sensing or CAD model-based approaches might seem like natural alternatives, they face significant barriers in real-world, high-speed production settings. Deploying accurate 3D inspection in-line is often hindered by high costs, sensitivity to industrial environmental interference, and computational complexity for real-time processing [7,8,9]. On the other hand, generating synthetic data from precise CAD models to overcome sample scarcity is challenged by the non-trivial domain gap between rendered images and real-world captures, as well as inherent geometric and appearance variations in manufactured parts that perfect CAD models do not capture [10,11].

Therefore, the core challenge for reliable defect detection of complex metal parts lies in how to transform the 3D defect recognition problem with strong spatial dependencies into a robust and deployable 2D vision solution, without relying on expensive 3D sensing or precise CAD models. This transformation faces the dual systemic challenges of “imaging interference” and “sample scarcity”.

Existing research often addresses only one of these problems in isolation. While a stream of studies primarily mitigates imaging interference through specialized hardware or sensor design, it frequently overlooks the scarcity of real defect data needed for training robust models [12]. Another significant body of work concentrates on overcoming sample scarcity by developing advanced data generation or synthesis algorithms [13,14], yet these methods often rely on idealized imaging assumptions, creating a gap between synthetically generated training data and real captured images affected by complex optical phenomena. This fragmentation of the “imaging-generation-detection” pipeline—where hardware-centric methods lack data adaptability, and data-centric methods lack physical grounding—makes it difficult for most solutions to form a complete and reliable system in real industrial scenarios. Although emerging approaches attempt to integrate multiple information sources to improve detection effectiveness [15,16], such methods are generally confined to fusing data under predetermined imaging conditions and fail to fundamentally co-optimize the stages of physical imaging, data generation, and detection. Consequently, designing a tightly synergistic framework that proactively integrates “imaging-generation-detection” to systematically tackle the dual challenges of imaging interference and sample scarcity remains an open and critical problem.

To address the imaging challenges posed by complex geometries and heterogeneous surfaces, researchers have attempted various hardware solutions. Fixed lighting schemes are prone to introducing uncertain reflections and shadows on curved surfaces. Gerges and Chen employed a large-scale LED array and convex optimization to enhance 2D recognition [17], while Wu et al. utilized dome lighting and photometric stereo to encode depth information [18]. These methods improve imaging quality but increase system complexity and cost, and do not solve the fundamental problem of sample scarcity. On the other hand, solutions represented by 3D sensing (e.g., structured light, laser scanning) or CAD model comparison, while capable of directly acquiring geometric information, face limitations for large-scale, high-speed production lines due to their high costs, complex on-site deployment and maintenance, sensitivity to highly reflective materials, and the misalignment between geometric reconstruction and the characterization of optical defects (e.g., fine scratches) [19,20]. These solutions collectively reflect a limitation in current research: the optimization of hardware imaging is often independent of downstream data generation and recognition tasks, lacking systematic design oriented towards the final detection performance.

At the data level, to alleviate the problem of sample scarcity, generative methods have garnered significant attention. Traditional data augmentation techniques struggle to preserve the morphological integrity of defects [21]. Copy–paste-based methods (e.g., CutPaste [22]) can change defect locations, but their fusion with the background is often unnatural; similarly, methods like NSA [23] improve boundary naturalness through Poisson image editing, but their core function remains redistributing existing defect patterns, making it difficult to create defects with entirely new morphologies or textures. Generative Adversarial Networks (GANs) demonstrate the potential for high-fidelity image synthesis [24], and works like Defect-GAN [25] and SDGAN [26] have shown the potential of GANs for generating defect images. However, the conventional GAN paradigm, which aims to directly generate complete defect images end-to-end from random noise, faces inherent limitations in complex industrial scenarios. This approach requires the generator to jointly and simultaneously model the intricate optical properties of the non-defective background and the subtle morphological features of the defect itself. This often leads to objective confusion and training instability, as the model struggles to decouple these two distinct learning tasks [27,28]; substantial computation is consumed on reconstructing the already adequate normal background, causing redundancy; complex backgrounds can severely interfere with defect generation, often leading to mode collapse. Algorithmic improvements for few-shot scenarios (e.g., FastGAN [29]) enhance training efficiency [30], but their static fusion mechanisms remain insufficient when dealing with complex geometries and heterogeneous textures. Consequently, dynamic multi-granularity feature fusion has become key to enhancing model representational capacity. For example, the Squeeze-and-Excitation (SE) module enhances feature sensitivity through channel-wise recalibration [31], but its single-resolution processing limits cross-scale adaptability; Residual Networks (ResNet) mitigate the vanishing gradient problem via skip connections [32] but lack a strategy for dynamic cross-layer fusion. Recent breakthroughs combine attention mechanisms [33] with Multi-Scale Fusion (MSF) [34] to achieve resolution-adaptive feature weighting, such as the CNN–Transformer parallel architecture for industrial image super-resolution [35], or multi-scale convolutional kernels with global feature selection [36]. These advances at the algorithmic level indicate that multi-granularity dynamic fusion is an effective way to improve a model’s ability to handle complex content.

However, a fundamental, system-level bottleneck persists: even with the introduction of more advanced dynamic fusion mechanisms, existing generative methods are still largely researched and validated based on relatively “clean” image data. They fail to be co-designed with the physical characteristics of the front-end imaging system (e.g., specialized lighting schemes designed to suppress highlights and shadows). When applied to images acquired from real industrial settings, which contain complex optical interferences (such as non-uniform reflections, strong shadows) that are difficult to completely strip away via algorithms, the generative capability of the models significantly degrades. Therefore, optimizing the generation algorithm in isolation while ignoring deep coupling with the front-end imaging cannot fundamentally solve the problem of defect sample generation in complex industrial scenarios. This deeply reveals the necessity of systematically integrating “imaging hardware optimization” and “data generation algorithms,” and bridging this gap is one of the core objectives of this work.

In summary, there is a clear fragmentation in current research across the three key stages of “imaging hardware, data generation, and defect recognition.” Hardware research does not fully consider providing optimal input for few-shot learning; generative algorithm research is often detached from real imaging physical constraints. This fragmentation makes it difficult for existing solutions to constitute a complete, reliable, usable, and deployable system under the harsh conditions of real industry (strong imaging interference, extremely low defect rates).

To overcome this systemic bottleneck, this paper proposes and implements a task-driven, end-to-end paradigm for constructing a defect detection system. The core idea of this work is, through the co-design of imaging physics, data synthesis, and detection models, to transform the 3D defect recognition problem for complex metal parts into a robust, deployable 2D vision solution. We no longer view imaging, generation, or detection in isolation, but integrate them into a closed-loop optimization process: the imaging subsystem provides “clean” input for data generation; the data generation engine, constrained by the characteristics of front-end imaging, efficiently synthesizes high-quality defect samples; finally, the performance of the downstream detector validates and provides feedback on the effectiveness of the entire system. The main contributions of this system are reflected in the following three closely related aspects:

(1) 3D-to-2D Defect Mapping Strategy: The core of this paper is proposing a systematic modeling method with a rigorous theoretical framework (Section 2.1). Through a systematic approach co-designing the imaging system and deep learning algorithms, along with methods adapted for highly reflective metal materials, this strategy uses 2D images as an intermediary to convert 3D defects into discriminable 2D features, achieving a stable mapping from 3D defects to 2D images. Differing from methods for conventional textured objects, this method targets metal parts with high curvature, complex structures, and directionally sensitive defects, balancing lightweight implementation and high detection performance without relying on 3D reconstruction, point clouds, or CAD models.

(2) Robust Imaging Subsystem for Highly Reflective Complex Workpieces: A dedicated imaging device integrating precision fixtures, dome diffuse lighting, and a small-aperture lens was developed. This subsystem effectively suppresses specular highlights and irregular shadows, providing high-quality images with uniform illumination and salient features for subsequent processing stages, reducing imaging interference at the source.

(3) Task-Driven Defect Imaging + Generation Software Architecture: With the ultimate goal of “training a few-shot detection model,” the first stage, based on an improved FastGAN, focuses on generating local defect patches. By reducing the dimensionality of the generation space, it effectively avoids interference from background textures and significantly reduces parameter count and computational cost. Simultaneously, a Dynamic Multi-Granularity Fusion (DMGF-SLE) module is introduced, which employs cross-layer channel and spatial attention gating mechanisms to achieve adaptive multi-scale feature fusion, thereby enhancing the representation capability for defect texture, shape, and structural anomalies. Furthermore, a perceptual loss is incorporated during training to improve the texture details and visual realism of the generated outputs. The second stage employs an optimized fusion mechanism based on the principles of Poisson image editing, which uses a designed key region mask to precisely control the location of defect generation. A composite loss function is also designed to ensure edge smoothness and geometric continuity, ultimately producing complete defect samples.

**Figure 1:**
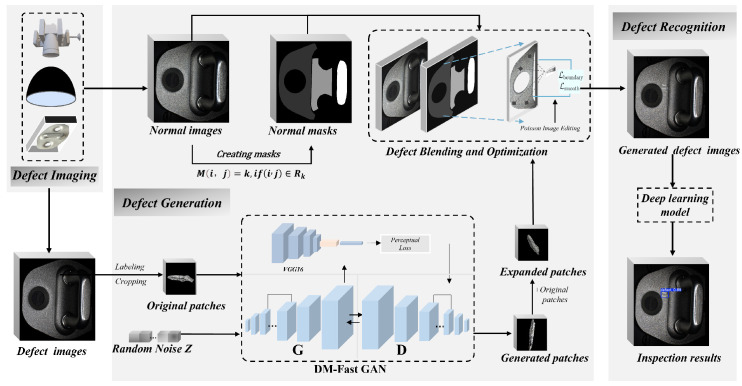
Overall workflow of the proposed defect inspection system. This process consists of three main stages: data acquisition using a dome light source and a small aperture (Defect Imaging), generating defect samples through a two-stage defect generation method (Defect Generation), and obtaining inspection results via a deep learning model (Defect Recognition).

**Figure 2:**
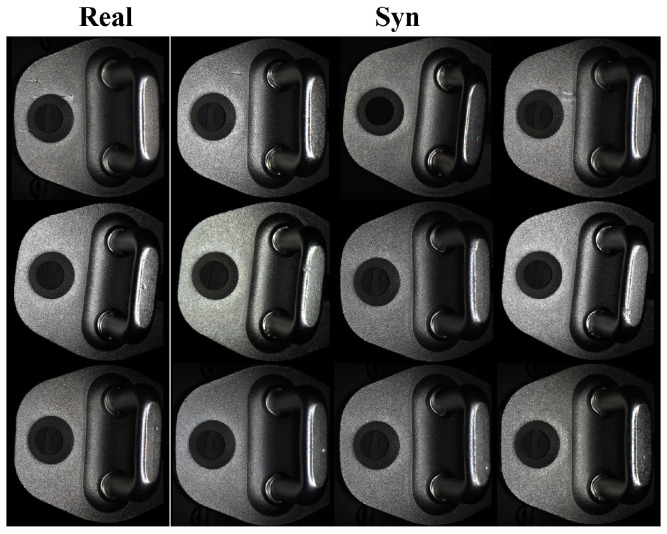
Comparison of real and generated defect images. Left column: real reference samples. Other columns: high-fidelity images generated by our method. Each row represents a category of defects: Linear (1st row), Compound (2nd row), and Punctate (3rd row). The results demonstrate that our method can generate sufficiently realistic and diverse training data for industrial inspection applications.

The overall workflow of the proposed system is illustrated in Figure 1. Initially, the imaging system is employed for data acquisition to obtain original defective and non-defective samples, which are then processed to yield key region masks for the normal samples and the original defect patches. Random noise is fed into the generator, which is focused on generating local defects, thereby mitigating the interference of complex optical features from the normal regions on the global synthesis process. A pre-trained VGG16 network [37] is introduced. By combining a perceptual loss function with the adversarial loss, the generator is optimized to produce defect patches of higher quality, enriched with optical details such as texture and illumination consistency. These generated defect patches are combined with real defect patches to form an expanded defect patch dataset, which is then paired with real normal images and their corresponding masks. Within the defect fusion and optimization module, the defect patches are fused onto the normal images at the designated defect locations guided by the masks. The fusion process employs Poisson image editing, a content loss, and a boundary smoothing loss to refine the blending result, ensuring that the generated defect regions retain high-quality details and exhibit natural transitions. These synthetic defective images are subsequently fed into a deep learning model for defect recognition, where the model analyzes the images to identify and localize defects, ultimately generating automated inspection results that complete the end-to-end defect detection pipeline. The final output is a complete synthetic defective image. Representative images generated by the proposed method are shown in Figure 2.

## 2. Materials and Methods

### 2.1. Theoretical Framework and Collaborative Imaging System Design

#### 2.1.1. 3D-to-2D Defect Mapping: Formulation and Co-Design Principles

This study employs the automotive door lock strike as the experimental subject. Its surface exhibits both complex textures and significant three-dimensional topographic features, making it a representative case for defect detection research on non-planar metal workpieces. Illuminating sheet metal assemblies like door lock strikes presents several challenges: their highly reflective surfaces are prone to specular reflections, causing glare and highlight regions that compromise imaging uniformity [38]; their complex geometries (e.g., curves, holes, and protruding edges) result in diverse light reflection paths, making uniform coverage difficult and prone to causing shadows or multiple reflection artifacts; furthermore, non-uniform lighting and ambient light interference further amplify brightness disparities in the images [39], adversely affecting the capture of defect details and the recognition accuracy of algorithms. These challenges necessitate optimizing the illumination and camera settings at the hardware level to enhance image quality and defect visibility. This system must be co-designed with downstream deep learning algorithms to realize a 3D-to-2D defect mapping strategy, thereby supporting lightweight AI model training based solely on high-quality 2D images without complex 3D modeling or projection correction. It is important to note that although the system is constructed under controlled conditions, its design goal is not to pursue idealized data, but to provide a unified, high-quality data reference baseline for defect detection algorithms, particularly during the GAN training phase, thereby enhancing model training stability. Practical industrial environments still exhibit varying degrees of lighting fluctuations and noise interference. Therefore, future work plans to incorporate multi-light source configurations and multi-environment sampling strategies to further enhance the model’s generalization capability and robustness under real-world conditions.

Building upon the imaging system design, we provide a rigorous mathematical formulation of the 3D-to-2D defect mapping strategy to address the relationship between imaging parameters and defect discernibility. In this work, the proposed 3D-to-2D defect mapping is defined in an operational and task-oriented manner, with the goal of establishing a stable and visibility-preserving correspondence between local 3D surface regions and their 2D image representations used for supervised defect detection. Let $S_{i}\subset R^{3}$ denote a local surface neighborhood on the inspected part, characterized by its local geometry (e.g., surface normal distribution and curvature). The 3D-to-2D defect mapping is formulated as a composition of the image formation process and the downstream task representation:

(1) $$M_{\theta }\left(S_{i}\right)=\psi \left(\Pi_{\theta }\left(S_{i}\right)\right),$$

where $\Pi_{\theta }\left(\cdot \right)$ represents the image formation operator parameterized by the imaging configuration θ, including illumination distribution, aperture (f-number), and admissible pose and curvature variations of the part, and ψ(·) denotes the 2D task representation used for defect detection, such as region-of-interest (ROI) or patch-level image features [40,41].

Under this formulation, a key objective of the proposed collaborative design is to ensure that variations in θ caused by surface curvature and part geometry do not introduce excessive instability or visibility loss in the resulting 2D representation. Specifically, for normal (non-defective) surface regions, the mapping $M_{\theta }\left(S_{i}\right)$ is expected to remain stable under admissible imaging variations, while for defective regions, the mapping should preserve sufficient contrast and structural detail for reliable detection.

Rather than introducing feature-space separability measures, which are redundant under fully supervised training, we characterize the quality of the 3D-to-2D mapping through physically interpretable imaging proxies that directly reflect defect visibility. In particular, the influence of imaging parameters is quantified by (i) a Laplacian-based sharpness metric, which measures the preservation of high-frequency defect structures and is sensitive to defocus, diffraction, and glare [42], and (ii) the background noise level (σ), which captures illumination- and geometry-induced intensity fluctuations caused by specular reflection and surface curvature [43,44]. Through these metrics, the relationship between imaging parameters and 2D defect visibility can be explicitly analyzed: illumination distribution primarily affects σ by suppressing or amplifying specular-induced background variations, aperture controls the trade-off between depth of field and diffraction-induced blur, and surface curvature influences both sharpness degradation and noise amplification through geometry-dependent reflectance [45]. The effectiveness of the resulting 3D-to-2D mapping is ultimately validated by supervised detection performance, demonstrating that the proposed collaborative design yields stable and discriminative 2D representations across complex 3D geometries.

#### 2.1.2. Hardware Implementation and Parametric Optimization

**Figure 3:**
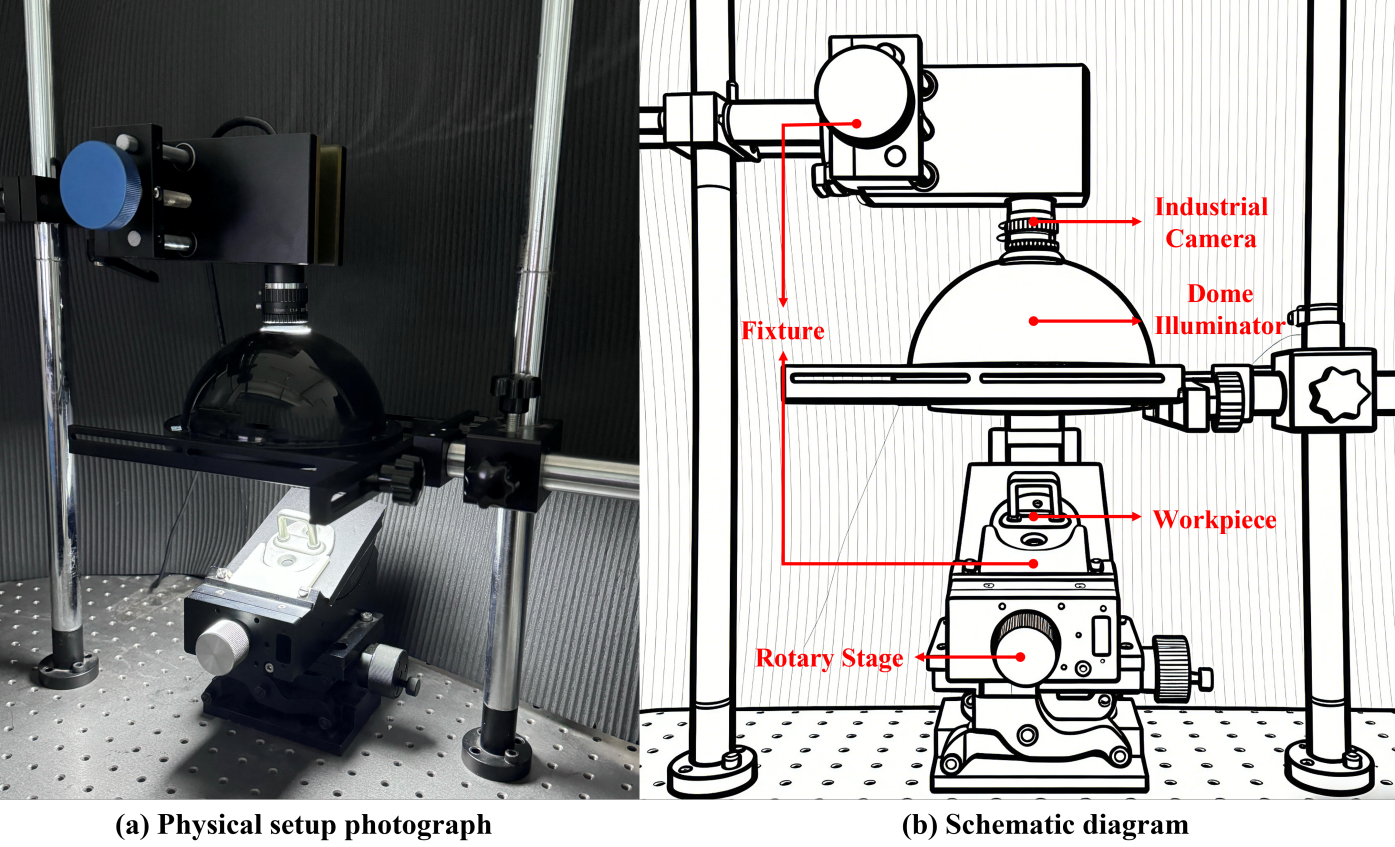
The experimental configuration of the imaging system utilizes a rotation stage and a positioning fixture to ensure precise control over the relative pose between the workpiece and the camera/light source. All components are rigidly mounted to an optical table via mounting rods to isolate vibration.

The imaging system, as shown in Figure 3, is configured at the hardware level into functional modules, primarily comprising three subsystems: image capture, illumination, and positioning. Specifically, the image capture module employs a Hikvision MV-CE120-10GC camera (Hangzhou Hikvision Digital Technology Co., Ltd., Hangzhou, China), coupled with a zoom lens (zlkz), with the image resolution set to 4024 × 3036 pixels to ensure the capture of minute defect details. The illumination module utilizes a custom-designed dome light source, which incorporates multiple sets of independently controllable LED arrays and achieves omnidirectional uniform diffuse lighting through an optical diffuser, significantly suppressing local specular reflections and irregular shadows. The positioning module comprises a high-precision rotation stage and a custom fixture. The workpiece is securely clamped onto the rotation stage, ensuring that during imaging, only rotational motion around a preset axis occurs, with no other degrees of freedom, thereby fundamentally eliminating translation and tilt errors. This hardware configuration is optimized based on preliminary experimental studies, with its parameters achieving a good balance among resolution, real-time performance, and robustness. It is specifically adapted for highly reflective materials, realizing a co-design methodology that integrates optics, imaging, and algorithms to address the challenge of high reflectivity on metal surfaces.

**Aperture Optimization Analysis.** The selection of a small aperture is closely related to the depth-of-field (DOF) requirements in industrial inspection. Under near-field imaging conditions, where the object occupies a non-negligible portion of the sensor field of view, the DOF can be approximated using the well-established macro-imaging formulation:

(2) $$DOF\approx \frac{2Nc(1+m)}{m^{2}}$$

where *N* is the f-number, *c* is the acceptable circle of confusion (CoC), and *m* is the magnification [46,47]. In our system, using the MV-CE120-10GC camera (Sony IMX226 CMOS sensor (Sony Corporation, Tokyo, Japan), resolution 4024×3036, pixel size 1.85μm×1.85μm), the magnification is determined from the calibrated object-space resolution: 1 pixel on the sensor corresponds to 17μm on the object, yielding m=1.85/17≈0.1088. The acceptable CoC is set according to the detection task: the final processed image (down-sampled to 1024×1024) has a scale of 1 pixel = 68μm on the object. To keep the defocus blur within 3 pixels in the final image (i.e., an object-space blur diameter of 0.204mm), the corresponding image-side CoC is c=0.204mm×0.1088=0.0222mm=22.2μm. The choice of 3 pixels for the blur tolerance (rather than the more typical 1–2 pixels) is conservative and task-driven, accounting for the combined effects of diffraction, residual defocus, and imaging noise under small-aperture operation.

With these parameters, the DOF for different apertures is:

(3) $$\begin{array}{cc} {\mathrm{DOF}}_{f/11} & \approx \frac{2\times 11\times 0.0222\times (1+0.1088)}{{\left(0.1088\right)}^{2}}\approx 45.7\;\mathrm{mm}, \end{array}$$

(4) $$\begin{array}{cc} {\mathrm{DOF}}_{f/16} & \approx \frac{2\times 16\times 0.0222\times (1+0.1088)}{{\left(0.1088\right)}^{2}}\approx 66.5\;\mathrm{mm}. \end{array}$$

The door-lock component has a geometric height of about 30 mm and is mounted with an inclination of approximately 22°. The focal plane is not set at the geometric mid-plane but is biased toward the base region, which is the primary inspection area. Consequently, the effective depth variation that must be covered by the DOF is the maximum axial deviation of surface points from the focal plane, $\Delta z_{\max}=\max\left|z\left(x,y\right)-z_{0}\right|\leq \mathrm{DOF}/2$, where $z_{0}$ is the height of focal plane. Measurements show $\Delta z_{\max}\approx 32\;\mathrm{mm}$, requiring a minimum DOF of about 64mm.

At f/11 the available DOF (≈45.7 mm) is insufficient to cover the full depth variation, risking defocus in the top and bottom regions. In contrast, f/16 provides a DOF of approximately 66.5mm, which comfortably exceeds the required 64mm and therefore offers a robust margin for assembly tolerances, part-to-part variations, and curvature changes.

To address the trade-offs in aperture selection, we performed quantitative DOF calculations, diffraction limit analysis (using Airy disk diameter), and signal-to-noise ratio (SNR) evaluation. For door lock components with varying curvature radii, the DOF requirements differ across areas: the top and bottom (smaller curvature radii, requiring larger DOF to cover protrusions and depth variations), and middle (higher curvature, needing precise focus with moderate DOF to avoid blur on transitions). A small aperture increases DOF to accommodate these variations but introduces diffraction blur and reduced SNR.

The diffraction limit is quantified by the Airy disk diameter [48,49]:

(5) $$d_{\mathrm{Airy}}=2.44\lambda N$$

where λ=0.55μm (average visible light wavelength). It is converted to pixels as follows: $d_{\mathrm{pixels}}=d_{\mathrm{Airy}}$/1.85 μm. SNR is proportional to $\sqrt{\mathrm{photons}}$, with photon flux $\propto 1/N^{2}$, so relative SNR ∝1/N (shot-noise dominant, assuming constant exposure via dome illumination compensation).

Theoretical results for f/11, f/16, and f/22 (with m≈0.109, c=22.2μm):

- f/11: DOF ≈45.7 mm (insufficient for the full curvature coverage, risking blur in top/bottom); Airy disk ≈14.76μm (7.98 pixels, high sharpness); Relative SNR ≈1.45 (strong for weak defects, minimal noise impact).
- f/16: DOF ≈66.5 mm (optimal balance, adequately covering curvature variations across top/middle/bottom without excess); Airy disk ≈21.47μm (11.61 pixels, acceptable sharpness for defects spanning tens of pixels); Relative SNR =1.00 (baseline, sufficient for weak defects with dome compensation).
- f/22: DOF ≈91.6 mm (excessive for needed coverage, but introduces significant blur); Airy disk ≈29.52μm (15.96 pixels, high diffraction-induced blur degrading edge details); Relative SNR ≈0.73 (reduced by ∼27%, potentially increasing false negatives for weak defects by 10–15% due to amplified noise).

These calculations confirm f/16 as optimal: it provides adequate DOF for the door lock’s varying curvature (top/bottom differences addressed by ∼66.5 mm coverage, middle precision maintained) while minimizing diffraction blur (Airy disk remains below thresholds where it dominates defect visibility, assuming defects >10 pixels). Smaller apertures like f/22 reduce SNR, adversely affecting weak defect visibility (e.g., shallow scratches with low contrast), as photon efficiency drops and noise amplifies under fixed exposure. Larger apertures like f/11 offer better SNR and sharpness but insufficient DOF, leading to out-of-focus regions on curved surfaces.

Rationale for the Combined Imaging Scheme. For non-planar metal components such as automotive door lock strikes, our system adopts an optimized solution combining industrial-grade dome lighting with small-aperture (f/16) imaging to address the challenges posed by high reflectivity and surface curvature variations. This scheme, serving as a core component of the 3D-to-2D defect mapping strategy, utilizes precision fixturing, dome-based diffuse illumination, and small-aperture lens imaging to deliver uniform lighting and enhance the saliency of structural features. It ensures high image contrast and detail integrity, improves defect controllability and the generalization performance of downstream detection, outperforming traditional point light source or large-aperture setups.

The dome light source, by providing omnidirectional uniform diffuse illumination, effectively eliminates glare and hotspots on highly reflective surfaces, reduces shadow interference, and ensures lighting consistency across curved, bent, and recessed areas [50]. Simultaneously, its uniform illumination subdues surface textural features (e.g., minor height variations caused by machining marks), reducing textural contrast and making them appear smooth in the image. In contrast, the asymmetry and significant morphological disturbances of defects (such as scratches, dents, or protrusions) disrupt the uniform light distribution, resulting in more pronounced local intensity variations, thereby enhancing defect visibility and contrast.

Complementing the dome light, the use of a relatively small aperture (f/16) was intentionally adopted to further improve imaging stability. While a reduced aperture introduces a certain degree of diffraction-induced blur and may slightly affect edge sharpness—representing a conscious trade-off in ultimate spatial resolution—it effectively suppresses unstable specular reflections that otherwise dominate the image under strong illumination. This configuration also extends the depth of field [51], ensuring the entire non-planar workpiece remains in sharp focus, and helps suppress stray light and avoid overexposure to preserve fine details [52]. From a defect inspection standpoint, this design prioritizes defect contrast and detection robustness over spatial resolution. The effectiveness of this trade-off is experimentally demonstrated in Section 3.2.

This combined scheme leverages the synergistic action between the multi-directional diffuse reflection of the dome source and the glare-suppressing property of the small aperture [53], achieving sensitive amplification of minute topographic perturbations: on normal surfaces, reflected intensity is weak and uniform; in defect regions, local normal disturbances cause reflected light from specific angles to intensify, forming bright or dark spots, thereby highlighting the defects. By employing this specific viewing angle and lighting scheme, the system ensures that defects exhibit consistent characteristics in the images, providing high-quality input for the subsequent two-stage generation method and supporting the task-driven generation architecture for 3D workpieces.

### 2.2. Controlled Data Acquisition Protocol

The detailed data acquisition workflow is as follows: This study employs a high-precision rotary stage to actively control the workpiece’s rotation about the *Z*-axis. First, the workpiece is secured in the fixture to ensure a consistent initial posture; subsequently, precise angular sampling is performed at 2.5° intervals within the range of 17° to 22°—a range determined based on common postural variations encountered in actual production lines. This specific narrow range was strictly defined by the physical constraints (e.g., fixture and vibration) observed on the partnered assembly line, and it also aligns with the anticipated pose deviation in future robotic grasping scenarios. Within this critical 5° fluctuation window, the 2.5° sampling interval represents an optimal engineering trade-off: it provides sufficient perspective diversity to simulate continuous pose changes crucial for model robustness, while avoiding the data redundancy that would arise from an excessively dense sampling scheme. After image acquisition for one side is completed, the workpiece is flipped and re-fixtured with precision, and the other side is captured following the same protocol to achieve comprehensive coverage of the workpiece surface. This controlled variation in viewing angle not only simulates reasonable pose fluctuations present in production lines but also enhances the diversity of defect manifestations and the comprehensiveness of the dataset. Furthermore, this controlled perspective variation, coupled with an image standardization and defect contrast enhancement mechanism, supports the stability of downstream GAN training.

### 2.3. Modifications to GAN for Optical Defect Imaging

#### 2.3.1. Dynamic Multi-Granularity Fusion Skip-Layer Excitation (DMGF-SLE) Module

Building upon the original FastGAN generator architecture, we introduce several enhancements, including an improved Skip-Layer Excitation (SLE) module and the integration of perceptual loss during training. The model adheres to the minimalist design philosophy of FastGAN, maintaining the classical adversarial training framework with two main components: the generator *G* and the discriminator *D*.

FastGAN incorporates the SLE module, which preserves gradient flow and feature recalibration through cross-resolution skip connections and channel-level multiplication, effectively reducing computational overhead. The SLE module is defined as:

(6) $$x_{\mathrm{enhanced}}=F\left(x_{\mathrm{low}},\left\{W_{i}\right\}\right)\cdot x_{\mathrm{high}}$$

where $x_{\mathrm{enhanced}}$, $x_{\mathrm{low}}$, and $x_{\mathrm{high}}$ represent the input and output feature maps of the SLE module. F comprises a set of fully connected layers and nonlinear activation functions, generating weighted coefficients for the high-resolution feature maps. $W_{i}$ denotes the weights of the fully connected layers. The generated weights $F(x_{\mathrm{low}},\left\{W_{i}\right\})$ are applied to the high-resolution feature map $x_{\mathrm{high}}$ via element-wise multiplication (·), achieving channel weighting.

While FastGAN utilizes SLEBlock for feature calibration, its static fusion strategy lacks dynamic weight adaptation to input variations. Notably, SLE’s exclusive reliance on channel attention disregards spatial pattern modeling, impairing texture continuity and edge alignment. Furthermore, the unidirectional low-to-high feature propagation fails to achieve intra-level multi-granularity fusion, resulting in underutilized mid-level semantics for complex scene generation.

**Figure 4:**
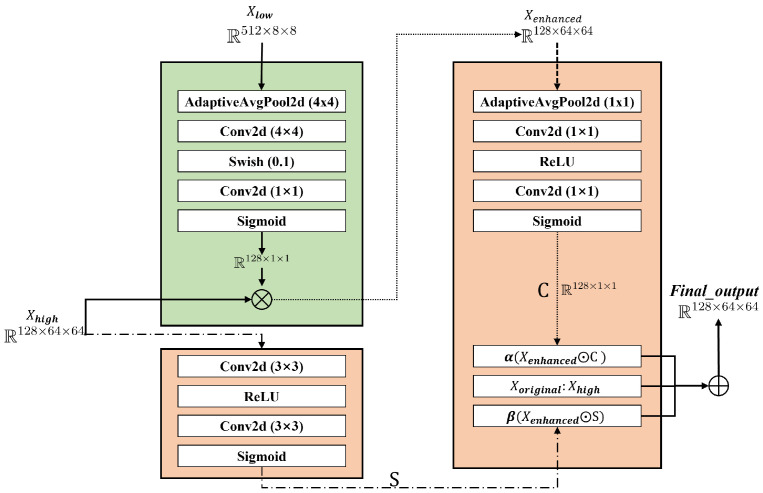
Structure of the DMGF-SLE module. This module combines the original SLE module and DMGF. The green box shows the processing path of the original SLE module, where low-level features undergo a series of convolutions and activation operations. The orange boxes represent the spatial and channel attention branches of DMGF, which work synergistically on input features. The enhanced features are fused with original features using learnable parameters alpha and beta.

To address the limitations of static or single-granularity feature fusion in existing attention mechanisms, we propose the Dynamic Multi-Granularity Fusion (DMGF) module. While previous works like DANet [54] and CBAM [55] employ learnable parameters, their design objectives are not tailored for the specific demands of multi-scale defect generation. For instance, DANet leverages self-attention to model long-range contextual dependencies for segmentation, but it lacks a mechanism for dynamic, input-dependent weighting across different resolutions. CBAM sequentially refines features through channel and spatial attention, yet it operates on a single feature scale and does not support multi-granularity fusion within a layer. In contrast, our DMGF module is explicitly designed with learnable parameters dedicated to adaptive, resolution-specific fusion tailored to GAN-based defect generation on non-planar surfaces. This enables dynamic balancing of defect complexities, such as varying scratch depths and surface normals, by prioritizing multi-scale semantics over static recalibration. Building upon SE module principles, DMGF enables adaptive cross-resolution fusion through dynamic weight adjustment. Drawing inspiration from Cao et al.’s dynamic feature fusion in object detection [56], our method emphasizes critical positional features via learnable attention mechanisms. Although sharing Flexible Parallel Fusion of CNN and Transformer (FPFCT)’s multi-scale fusion philosophy [35], DMGF introduces enhanced flexibility through dual-branch spatial-channel attention (Figure 4). This architecture dynamically adapts fusion strategies to input characteristics, captures multi-granularity patterns across and within layers, and mitigates over-reliance on enhanced features. By balancing semantic utilization with feature diversity, the module significantly advances complex image generation [57,58,59].

The DMGF module dynamically modulates feature fusion through synergistic channel-spatial attention, governed by:

(7) $$y=x_{\mathrm{high}}+\alpha \cdot \left(c\cdot x_{\mathrm{enhanced}}\right)+\beta \cdot \left(s\cdot x_{\mathrm{enhanced}}\right)$$

where α and β are direct learnable parameters (no activation constraints) controlling the influence of channel attention *c* and spatial attention *s* on the enhanced feature $x_{\mathrm{enhanced}}$. These parameters are initialized to 0.5 and optimized via Adam (learning rate 1 × 10^−4^). This adaptive adjustment enables the DMGF module to select the optimal fusion strategy based on input feature complexity. The attention mechanisms are defined as:

(8) $$c=\sigma \left(G_{\mathrm{channel}}\left(x\right)\right),\;s=\sigma \left(G_{\mathrm{spatial}}\left(x\right)\right)$$

where $G_{\mathrm{channel}}\left(x\right)$ computes channel attention through adaptive global average pooling followed by two 1×1 convolution layers (with channel reduction ratio 16), ReLU activation, and a final 1×1 convolution, and $G_{\mathrm{spatial}}\left(x\right)$ learns spatial correlations via two sequential 3×3 convolution layers with ReLU activation. Both attention branches terminate with Sigmoid activation (σ) to generate normalized attention maps.

To validate the dynamic nature of α and β, we monitored their values during training across resolutions (64, 128, 256, 512). Training logs (Table 1) show independent evolution from initial values ∼0.5 (Iteration 0: $\alpha_{64}=0.49995$, $\alpha_{128}=0.50005$) to task-specific optima ($\alpha_{64}=0.81715$, $\alpha_{128}=0.70642$ by Iteration 100,000), confirming backpropagation-driven adaptation without activation constraints.

Parameter Symmetry and Dynamic Validation: While both α and β are essential components, our training logs primarily capture the evolution of α parameters due to monitoring constraints. This is justified by: (1) Structural Symmetry: α and β share identical mathematical properties (direct learnable parameters, Adam-optimized) and symmetric roles; (2) Empirical Evidence: α shows clear adaptive behavior across resolutions (Table 1); (3) System-Level Validation: The performance advantage of dynamic fusion provides independent confirmation.

Channel attention weights global statistics (e.g., high-frequency edges in metal defects), while spatial attention activates local regions (e.g., glass scratches). The multi-resolution generator branches (64 × 64 to 1024 × 1024) adaptively adjust feature fusion: low-resolution layers preserve details, high-resolution layers enhance semantics, enabling scalable resolution adaptation without structural modification. In our implementation, we added DMGF-SLE modules at multiple levels (DMGF-SLE 64, 128, 256) to handle defect patches of different scales.

#### 2.3.2. Perceptual Loss

Perceptual loss [60] enhances generative models by comparing feature-space similarities between generated and real images through pre-trained CNNs (e.g., VGG). This mechanism measures structural/textural discrepancies in high-level representations rather than pixel-level differences, effectively enhancing visual realism while mitigating blur and artifacts [61,62,63].

**Figure 5:**
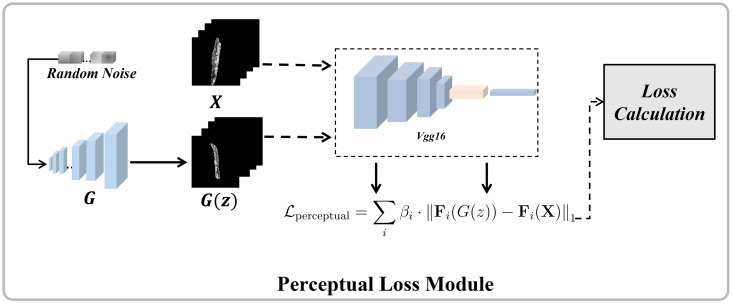
Perceptual loss. The deep image features extracted through the perceptual loss using the VGG network have stronger abstraction abilities and richer semantic information, which helps to improve the quality and realism of the generated images.

The perceptual loss module compensates for the original FastGAN’s shortcomings in capturing high-level features. As shown in Figure 5, we introduce a perceptual loss module during training, extracting deep features from the high layers of the VGG network to optimize the generation effect. Our loss function is defined as:

(9) $$L_{\mathrm{perc}}=\sum_{i}\beta_{i}\cdot {∥F_{i}\left(G\left(z\right)\right)-F_{i}\left(X\right)∥}_{1}$$

Here, *X* is the real image, *z* is the random noise, G(z) is the generated image, $F_{i}\left(\cdot \right)$ represents the feature map of the *i*-th layer of the pre-trained VGG network, and $\beta_{i}$ is the weighting coefficient used to balance the contributions of different feature layers. As shown in Figure 4, the module uses VGG to minimize the feature differences between *X* and the features extracted from G(z).

We adapted the architecture based on the design principles of Ledig et al. [64] and Zhang et al. [61], which eliminates the batch normalization (BN) layer and removes the fully connected layers. This modification allows the network to handle input images of varying sizes. We utilize the feature map from the fourth pooling layer of the fifth convolutional block as the output. This convolutional block, with its deep structure, is particularly effective at capturing high-level semantic features of images, making it well-suited for tasks requiring detailed visual representation. Through these improvements, we have significantly enhanced the generator’s performance in image generation tasks, improving the details and overall consistency of generated images while maintaining the model’s computational efficiency and training stability. These optimizations make the improved FastGAN perform excellently in generating high-quality, high-resolution images, suitable for a wider range of application scenarios.

**Figure 6:**
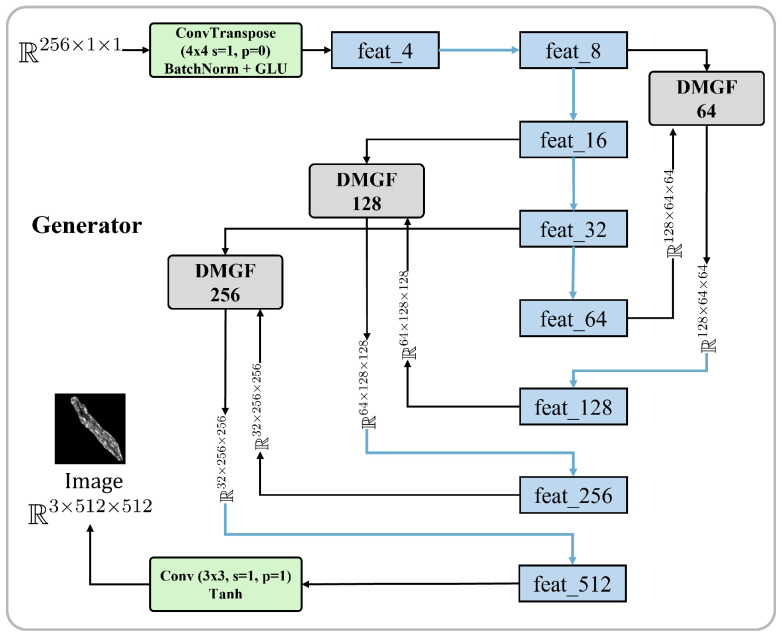
The structure of the generator of DMGF-FastGAN. The blue boxes in the figure represent feature maps (channel numbers omitted), the blue arrows indicate the same upsampling operation, and the gray boxes represent the DMGF-SLE module.

The generator *G* (Figure 6) processes noise vectors through initial deconvolution and multi-stage upsampling via nearest-neighbor interpolation with convolution-BatchNorm-GLU operations. The DMGF-SLE module activates at critical layers for cross-resolution fusion:

(10) $$X_{\mathrm{enhanced}}=X_{\mathrm{high}}⊙\sigma \left(\mathrm{Conv}\left(\phi \left(\mathrm{AdaptiveAvgPool}2d\left(X_{\mathrm{low}}\right)\right)\right)\right)$$

where $X_{\mathrm{low}}$ and $X_{\mathrm{high}}$ denote multi-scale features, ϕ represents Swish activation, and σ is Sigmoid. Dual attention mechanisms (channel/spatial) subsequently adaptively weight the fused features before final convolution layers generate outputs. This design enables hierarchical feature enhancement while maintaining computational efficiency.

### 2.4. Defect Blending and Optimization

**Figure 7:**
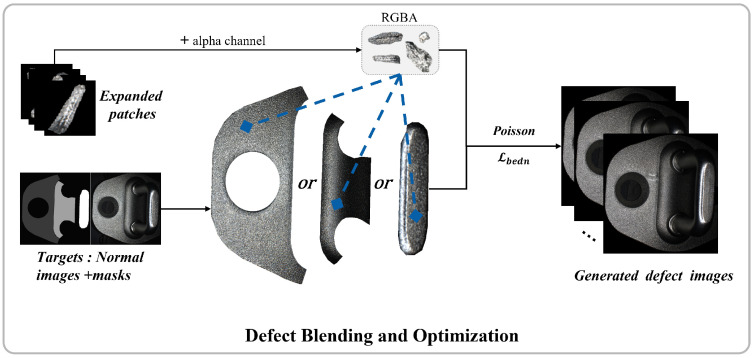
The process of Defect Blending and Optimization. Light blue arrows indicate the selection of different key regions as the approximate range of the defects.

As shown in Figure 7, the core of this methodology involves defect patch transplantation into normal samples via Poisson image editing [65] and customized loss functions. The workflow comprises: (1) Transparent channel conversion of defect patches, (2) Mask-guided positioning in key target regions, (3) Gradient-aware fusion through Poisson editing. This process simultaneously generates defect region masks (mask_c) and boundary masks (mask_boundary) to constrain the optimization domain. Our fusion loss function drives Adam-optimized iterative refinement, achieving visually seamless integration while preserving original defect characteristics.

By analyzing the relationship between defects and their positions in defect samples, a key region mask for the target image was designed, as follows:

(11) $$M_{i,j}=\left\{\begin{array}{cc} 50, & \mathrm{if}\;\left(i,j\right)\in R_{\mathrm{bottle}}\\ 150, & \mathrm{if}\;\left(i,j\right)\in R_{\mathrm{middle}}\\ 250, & \mathrm{if}\;\left(i,j\right)\in R_{\mathrm{top}} \end{array}\right.$$

Here, *i* and *j* represent the horizontal and vertical coordinates of a point in the image, respectively, and $M_{i,j}$ denotes the pixel value at that point. Through this concise design, the range of defect positions can be precisely controlled, and in combination with subsequent steps, it enables the generation of defects with diverse locations.

The loss is a weighted composite loss function. It consists of parts for smoothing boundaries and maintaining the original information of the defect areas:

(12) $$\begin{array}{cc} L_{\mathrm{bedn}} & =\;\beta \cdot \frac{1}{N}\sum_{i=1}^{N}H_{x}\left(i\right)\cdot M_{\mathrm{boundary}}\left(i\right)\\ \; & \;+\;\frac{1}{N}\sum_{i=1}^{N}H_{y}\left(i\right)\cdot M_{\mathrm{boundary}}\left(i\right)\\ \; & \;+\;\gamma \cdot \mathrm{MSE}(I_{\mathrm{defected}}\cdot M_{c},I_{\mathrm{original}}\cdot M_{c}) \end{array}$$

In the equation, $H_{x}\left(i\right)$ and $H_{y}\left(i\right)$ represent the gradients of the image in the horizontal and vertical directions, $M_{\mathrm{boundary}}\left(i\right)$ is the boundary mask, $I_{\mathrm{original}}$ is the original defect patch, $I_{\mathrm{defected}}$ is the optimized image, $M_{c}$ is the defect area mask, and β and γ are weighting coefficients. Boundary smoothing aims to alleviate abrupt changes and artifacts at the edges of generated images during the image restoration process. This part forces the boundary transitions of the repaired regions to be more natural and continuous by introducing gradient-based constraints. By applying edge detection filters similar to the Sobel operator to calculate the local gradients of each channel and combining them with the boundary mask $M_{\mathrm{boundary}}$, the focus on boundary regions is strengthened. Content preservation aims to avoid significant deviations between the repaired image and the original image, especially in areas with complex textures or fine structures. By calculating the Mean Squared Error (MSE) [66] between the repaired image and the original image in the defect areas, and using a mask to limit the focus to these areas, we ensure that comparisons are made only in the defect regions. By precisely adjusting the weights, the optimization process seeks a balance between content preservation and boundary smoothing, reducing abrupt breaks or unnatural jumps in boundary areas while maintaining the original information in defect regions. This study efficiently generates defect samples through a sophisticated combination by using a minimalist FastGAN architectural design, designing a new DMGF-SLE module, and introducing perceptual loss to generate defect patches. Simple image processing methods are employed to fuse defect patches into normal samples to create complete defect samples. This study balances the quality of generated defect samples with computational resource consumption.

## 3. Experiments

To validate the effectiveness of the proposed method, systematic experiments were conducted on an automotive door lock strike dataset, encompassing imaging quality assessment, defect patch generation, complete defect sample fusion, object detection performance improvement, and comparative analysis.

### 3.1. Experimental Setup: Dataset, Calibration, and Configuration

**Dataset and Annotation.** The dataset comprises 864 images of non-defective samples and 566 images of defective samples. The irregular defect regions on the defective samples were annotated using LabelMe (v3.16.7). These annotated regions were cropped and placed onto a pure black background to isolate the optical characteristics of the defects. The defects were categorized based on their type (linear, punctate, compound), with concentrations primarily in the top, middle, and bottom regions of the samples, showing a relatively fixed spatial distribution: linear defects were predominantly located in the bottom and middle areas, while punctate defects were mostly distributed in the top region. Some ambiguous defects were classified as “compound defects.” Given the relatively consistent locations of the defects, the complete door lock strike samples were further segmented into different regions. Mask images for these key regions were generated using LabelMe to guide the defect generation process based on optical imaging properties.

**System Calibration and Physical-Pixel Scale.** Our imaging system was calibrated using a high-precision calibration board to establish the physical-pixel correspondence. The original images captured at 4024×3036 resolution have a physical scale of 1 pixel = 17 μm (or 3000 pixels = 50 mm). After image processing (padding to 4096×4096 and downsampling to 1024×1024), the final working resolution corresponds to 1 pixel = 68 μm (0.068 mm) or 14.7 pixels/mm. This calibration was verified using multiple reference measurements across the image field to ensure accuracy.

**Defect Size Distribution and Statistics.** We conducted a comprehensive analysis of the physical dimensions of all defect samples. As shown in Table 2, the dataset exhibits a diverse range of defect sizes. The minimum detectable defect size is 0.73 × 0.57 mm (10.9 × 8.5 pixels), which is below the 1.0 mm threshold specified in automotive quality standards ISO 286-2 [67], validating the high sensitivity of our inspection system. The maximum defect size measured is 9.13 × 3.60 mm (137.0 × 54.0 pixels), with an average equivalent diameter of 2.28 ± 1.17 mm. This distribution reflects real-world industrial defect scenarios where medium-sized defects are most common, while extremely small or large defects occur less frequently.

**Model Training Configuration.** The Generative Adversarial Networks (GANs) were trained on an RTX 3080 (20 GB) GPU (NVIDIA Corporation, Santa Clara, CA, USA) using PyTorch 2.0. We adopted a “divide-and-conquer” strategy by training separate GAN models for each defect category. This ensures that each model focuses exclusively on learning the data distribution of a specific defect pattern, thereby enhancing the diversity and representativeness of the generated samples. During training, separate models were developed for each defect category with input sizes and batch sizes configured according to the specific defect characteristics, as detailed in Table 2.

### 3.2. Imaging Quality Assessment Experiment

To systematically validate the effectiveness of the proposed imaging scheme on complex metal workpieces, this study conducted a comprehensive comparative analysis across visual quality, objective image metrics, and downstream detection performance and robustness. A 2 × 2 factorial design was employed to deconstruct the individual and combined contributions of the two core design principles: uniform dome illumination versus directional lighting, and a small aperture (large depth of field) versus a large aperture. Four representative optical configurations were selected: Dome+f/16 (our proposal), Ring+f/16 (ring light + small aperture), Bar+f/16 (bar light + small aperture), and Dome+f/4 (dome light + large aperture). All images were captured from the same batch of automotive door lock strike workpieces under strictly controlled, fixed working distances and illumination intensities. Image quality was assessed using sharpness (Laplacian gradient norm) and noise level (image standard deviation σ). Downstream performance was evaluated using a YOLOv8 model, measuring mAP@50, mAP@50:95, F1-Score, and critically, the average number of false positives per image (FPs/img) to quantify detection robustness under identical training and validation settings.

**Figure 8:**
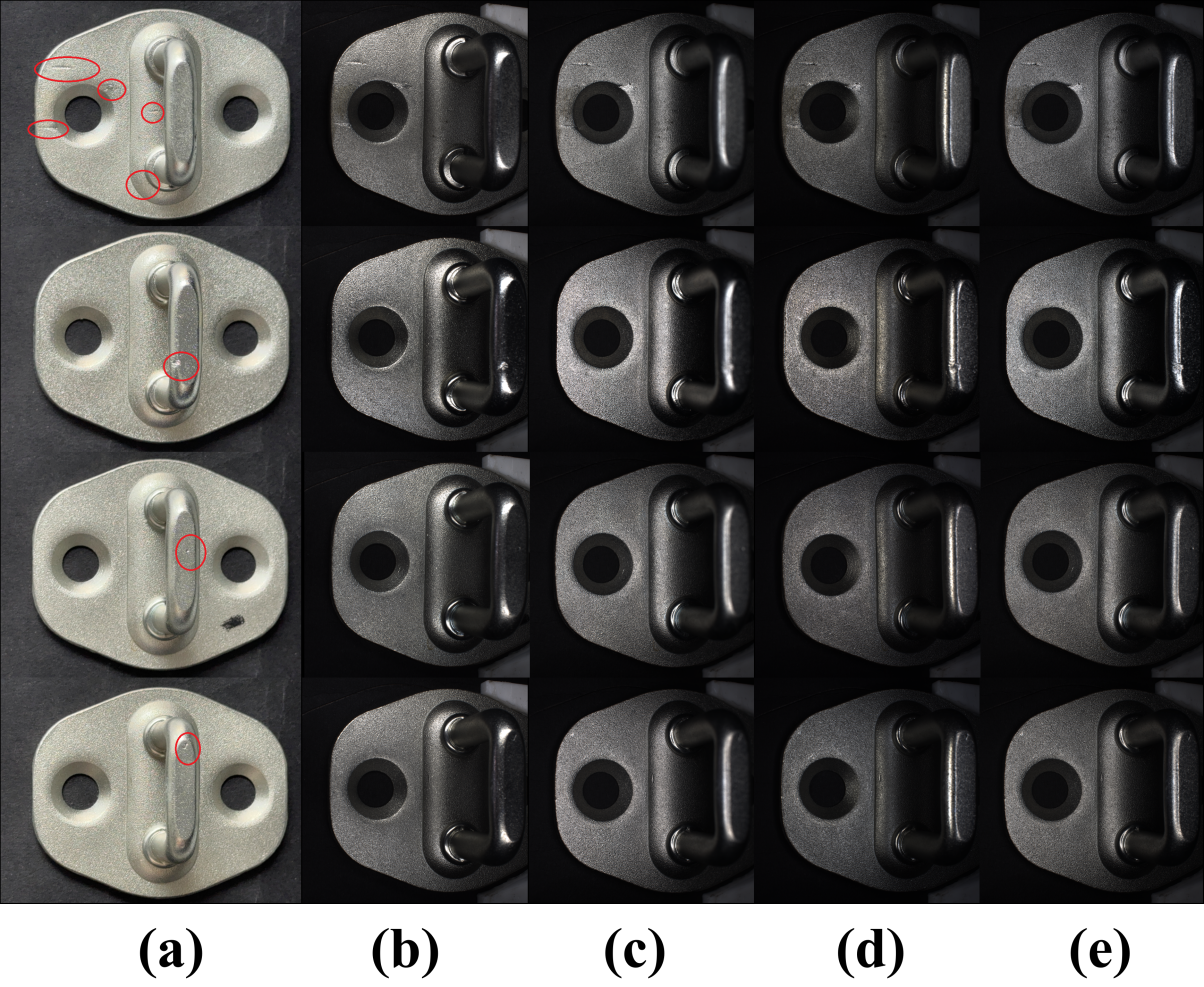
Defect imaging results on the same metal workpiece surface under five configurations. (**a**) Mobile phone with indoor lighting, (**b**) Bar light + small aperture, (**c**) Dome light + large aperture, (**d**) Ring light + small aperture, (**e**) Our method (Dome light + small aperture): Combining isotropic uniform irradiation and a large depth of field, it comprehensively suppresses reflections/shadows, achieves clear imaging across the entire field of view, smoothens background texture with a uniform light field, and highlights defects as high-contrast features due to scattering anomalies.

As shown in Figure 8, the visual comparative analysis first qualitatively demonstrates the effect differences. (a) Mobile phone with indoor lighting exhibits low contrast and blurred edges. (b) Bar light + small aperture enhances local contrast but introduces strong directional reflections and overexposure. (c) Dome light + large aperture suppresses highlights but suffers from a shallow depth of field, preventing full-field sharpness on curved surfaces. (d) Ring light + small aperture offers a balance but retains top reflections that reduce local contrast. (e) Our method (Dome light + small aperture) effectively suppresses reflections and shadows across the entire field of view, resulting in a smooth background where defects are highlighted as high-contrast features, confirming the necessity of co-designing “uniform illumination” and “large depth of field.”

#### 3.2.1. Experimental Validation of Aperture Selection

To further validate the aperture selection, we conducted ablation experiments under identical dome illumination and exposure-matched conditions. The experiments used the same door-lock components, captured images with f/11, f/16, and f/22 apertures respectively, and employed the same YOLOv8 model for defect detection, with mAP@50 as the evaluation metric.

The experimental results show that f/11, due to insufficient DOF, caused local defocus on curved surfaces, reducing defect sharpness and detection performance. Although f/22 provided sufficient DOF, diffraction blur and reduced SNR degraded image quality, also affecting defect detection accuracy. f/16 maintained adequate DOF while controlling diffraction blur and noise, achieving the best detection performance. Quantitative results are summarized in Table 3.

Although a smaller aperture reduces light throughput, controlled dome illumination and exposure compensation prevent excessive noise amplification. Consequently, the SNR remains sufficient for weak defect detection, as reflected by the stable mAP@50 and low false-positive rates achieved with the f/16 configuration.

While reducing the aperture increases the depth of field, it also introduces diffraction-related blur. The diffraction-limited blur size can be estimated by the Airy disk diameter, $d_{\mathrm{Airy}}\approx 2.44\lambda N$. For white-light imaging, taking λ≈0.55μm, the corresponding Airy disk diameters for f/11, f/16, and f/22 are approximately 14.8μm, 21.5μm, and 29.5μm, respectively, corresponding to about 8.0, 11.6, and 16.0 pixels on the employed sensor. In our application, the characteristic spatial extent of typical surface defects (e.g., scratches and small surface irregularities spanning tens of pixels) remains significantly larger than the diffraction blur at f/16, indicating that diffraction is not the dominant resolution-limiting factor under this setting.

However, further stopping down to f/22 results in increased diffraction blur and reduced photon efficiency, which may adversely affect weak defect visibility. Therefore, f/16 represents a practical balance between depth-of-field coverage and diffraction-induced resolution loss.

#### 3.2.2. Comprehensive Performance Comparison

At the image quality level, significant differences are observed (Table 4). The Ring+f/16 configuration achieved the highest sharpness (303.70) due to directional edge enhancement, but at the cost of the highest noise level (3.71). In contrast, our Dome+f/16 scheme maintained excellent sharpness (266.40) while achieving the lowest noise (2.50), demonstrating effective speckle noise suppression. The Dome+f/4 result is critical: although using the same uniform illumination, its large aperture causes a severe sharpness drop (131.68), directly proving the necessity of a “small aperture” for full-field sharpness. It is important to clarify the intentional trade-off here: the relatively small aperture (f/16) was selected to ensure a large depth of field. While a reduced aperture may introduce slight diffraction-induced blur at the theoretical resolution limit, this choice prioritizes full-field focus and, crucially, suppresses unstable specular reflections on highly reflective surfaces. For defect detection, maximizing stable defect-to-background contrast and robustness is paramount over ultimate spatial resolution.

These fundamental differences in image quality directly and consistently translated to downstream defect detection performance and, most notably, robustness (Table 4). The model trained on Dome+f/16 data achieved the best overall performance, particularly on the comprehensive metric mAP@50:95 (50.4%) and F1-Score (0.89). The Ring+f/16 configuration, leveraging its high sharpness, achieved the second-best mAP@50:95 (47.1%). However, a decisive advantage of our proposed scheme is revealed in the false positive analysis. The Ring+f/16 configuration produced the highest rate of false alarms (2.3 FPs/img), nearly triple that of our method (0.7 FPs/img). This indicates that the directional highlights from the ring light, while enhancing some edges, create numerous spurious, defect-like features that degrade model reliability. The Bar+f/16 configuration, with the most unstable lighting, performed worst across all detection metrics, including false positives (3.0 FPs/img). Conversely, the isotropic diffuse light from the dome source provides a consistent, low-noise background, drastically reducing false alarms. The comparison between Dome+f/4 and Dome+f/16 further isolates the aperture’s role: the large aperture variant, despite its uniform lighting, suffers in overall detection performance (mAP@50:95: 47.7%) due to poor sharpness, though it maintains a moderate false positive rate (1.5 FPs/img) thanks to its stable illumination. This systematic ablation confirms that uniform dome illumination is the key to minimizing false positives and ensuring robustness, while the small aperture is foundational for achieving the full-field sharpness required for high detection accuracy. The proposed Dome+f/16 configuration optimally balances these factors, delivering superior performance where it matters most for industrial inspection: high detection accuracy coupled with high operational reliability.

### 3.3. Validation of the Effectiveness of the DMGF-SLE Module and Perceptual Loss

#### 3.3.1. Ablation Study on Core Modules: DMGF-SLE and Perceptual Loss

To evaluate the effectiveness of the DMGF-SLE module and perceptual loss in the first stage of our defect sample generation framework, we used the original FastGAN as the baseline and conducted experiments with three variants: FastGAN+DMGF, FastGAN+PER, and FastGAN+All (combining both DMGF-SLE and perceptual loss). Training was performed on an RTX 3080 GPU using the same defect patch dataset for 4 h per variant, with three random seeds to ensure reproducibility. We conducted ablation experiments on defect patches using FID (Fréchet Inception Distance) [68], LPIPS (Learned Perceptual Image Patch Similarity) [69], and IS (Inception Score) [70] as evaluation metrics. All experiments were conducted with multiple runs, and the mean values are reported to evaluate performance. FID quantifies feature distribution differences between real and generated images:

(13) $$\mathrm{FID}=∥\mu_{r}-\mu_{g}{∥}_{2}^{2}+\mathrm{Tr}\left(\Sigma_{r}+\Sigma_{g}-2{\left(\Sigma_{r}\Sigma_{g}\right)}^{1/2}\right)$$

Here, Tr denotes the trace of a matrix, and $\mu_{r},\Sigma_{r}$ and $\mu_{g},\Sigma_{g}$ represent the mean and covariance matrices of the real image data and generated image dataset in the feature space, respectively. LPIPS measures perceptual similarity via weighted feature distances:

(14) $$\mathrm{LPIPS}\left(x,y\right)=\sum_{l}w_{l}\cdot {∥\phi_{l}\left(x\right)-\phi_{l}\left(y\right)∥}_{2}$$

Here, *x* and *y* are the two images to be compared, $\phi_{l}\left(\cdot \right)$ represents the feature maps extracted by the pre-trained deep network (such as AlexNet [71] or VGG at the *l*-th layer, and $w_{l}$ is the weighting coefficient used to balance the contributions of different feature layers.IS assesses diversity using the entropy of category distributions:

(15) $$\mathrm{IS}=\exp\left(E_{x∼D_{g}}\mathrm{KL}\left(p\left(y|x\right)\|p\left(y\right)\right)\right)$$

**Figure 9:**
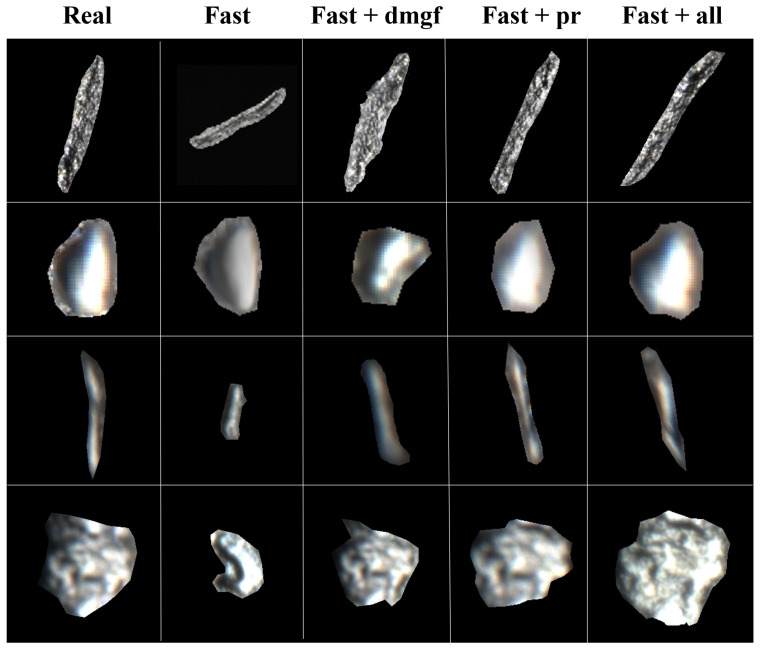
Ablation study results of different generation methods. The first column shows real defect patches, and each row represents defect patches of the same type generated by different methods. Our comparison reveals that the combination of the DMGF-SLE module and perceptual loss significantly enhances detail capture, structural consistency, and the overall quality of the generated images.

Here, *x* represents the generated image, p(y|x) denotes the category probability distribution predicted by the classification network for the generated image *x*, and p(y) represents the average category probability distribution of the generated image dataset. Results in Table 5 demonstrate that adding DMGF-SLE or perceptual loss individually reduces FID, with the combined approach (FastGAN+All) achieving the lowest FID (e.g., 46.25 for linear defects), alongside lower LPIPS and higher IS scores, indicating enhanced quality, perceptual similarity, and diversity in defect patch generation, reflecting improved detail capture and variety in the generated patches. We also performed a visual evaluation conducted by professional workers from the production line to compare the generated defect patches across different methods under the same number of iterations, as shown in Figure 9. The first column displays real defect patches as reference images, while the other columns present defect patches generated by different methods, with each row corresponding to a specific defect type. When using FastGAN alone, the generated defect patches exhibit darker tones, simpler shapes and blurred edges, deviating significantly from real defect appearances. When DMGF-SLE is added alone, cross-layer feature interactions enhance the structure and texture details, resulting in sharper edges, though some unnatural jagged edges appear, particularly in the first and fourth rows of the third column. When only the perceptual loss module is used, it refines details by eliminating jagged edges and improving texture and structure, but the shapes tend to become more conservative. When both DMGF-SLE and perceptual loss are combined (FastGAN+All), the generated defect patches show significant improvements in detail capture, structural consistency, and natural transitions, closely resembling real defect patches in terms of texture, edge clarity, and overall visual quality.

#### 3.3.2. In-Depth Analysis of the Dynamic Fusion Mechanism

To validate the effectiveness of our dynamic fusion mechanism, we conduct comprehensive ablation studies comparing three variants: Baseline (Original FastGAN without DMGF-SLE module), Static (DMGF-SLE with fixed parameters (α=β=0.5)) and Dynamic (Full DMGF-SLE with learned parameters).

**Figure 10:**
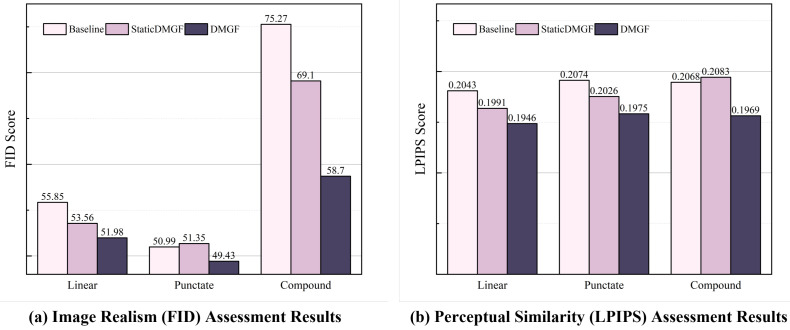
Performance Comparison of the DMGF-SLE Module and Its Variants on the Defect Generation Task.

The ablation results, as visually summarized in Figure 10, demonstrate that our dynamic fusion mechanism achieves significant and consistent improvements over both the baseline and static variants. The bar charts clearly show that the **Dynamic** variant (dark purple) achieves the lowest bar (best score) for every defect type in both FID and LPIPS metrics. Quantitatively, compared to the Baseline, the Dynamic variant reduces the FID by 6.93% for linear defects, 3.06% for punctate defects, and a substantial 22.01% for compound defects. Concurrently, it lowers the LPIPS by approximately 4.8% consistently across all defect types. This indicates a clear and simultaneous enhancement in both the realism (FID) and perceptual quality (LPIPS) of the generated patches. More importantly, the Dynamic variant consistently outperforms the Static variant. The visual gap between the medium pink (Static) and dark purple (Dynamic) bars is evident across nearly all categories. For instance, in the most challenging compound defects task, the Dynamic variant achieves a 15.1% lower FID (58.7 vs. 69.10) and a 5.5% lower LPIPS (0.1969 vs. 0.2083) than the Static variant, which is directly observable in the rightmost set of bars in both charts.

This consistent performance gap, now made intuitive by the comparative bar charts, provides strong evidence that both α and β parameters are actively and beneficially adapting during training. If the parameters were not effectively learning, the dynamic version would not reliably surpass the statically tuned version across all scenarios as shown. The progressive adaptation of these parameters (evident in Table 1) correlates directly with this improved generation quality, validating our design hypothesis that dynamic multi-granularity fusion is superior to a fixed fusion strategy, especially for handling complex and irregular defect patterns.

#### 3.3.3. Cross-Dataset Generalization Validation

**Figure 11:**
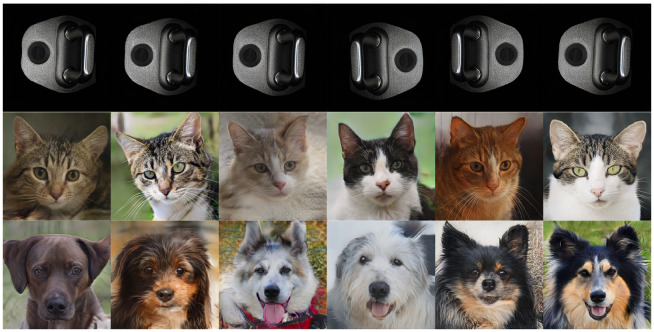
Images generated by training on different datasets using our method.

To further assess the generalizability of our improvements across diverse datasets, we evaluated FID on randomly selected images from the AFHQ_v2 (Animal Faces-HQ) [72] dog and cat categories and our car door latch dataset. Results and setups are presented in Table 6, with generated images shown in Figure 11. The consistent FID reduction across datasets (e.g., from 46.19 to 34.26 for the Car Door Latches dataset) validates the robustness of DMGF-SLE in enhancing defect patch generation for various applications.

### 3.4. Quality Evaluation Experiments Based on Defect Sample

To verify the authenticity of integrating generated defect patches into normal samples in the second stage, we conducted a quality comparison experiment. Since car door latch defects are small and easily obscured by similar non-defective regions, we expanded the defect areas into central patches. Given defect types vary by location, we selected top, middle, and bottom positions for patching. Three methods were tested—CutPaste, Poisson image editing, and our proposed approach—using the same defect patch, with quality assessed via FID (Fréchet Inception Distance).

Table 7 shows CutPaste yielding the highest FID scores (e.g., 160.98 at bottom), due to poor fusion with the background, reducing sample authenticity. Poisson and Ours achieve much lower FID scores, with Ours outperforming Poisson at top (25.34 vs. 28.32) and bottom (54.73 vs. 60.57) positions, and comparable performance at middle (33.63 vs. 34.78). This reflects our method’s ability to balance defect embedding and feature preservation in complex backgrounds. This is because our method not only considers embedding the defect block into the target sample but also emphasizes preserving the features of the defect region. This suggests that our method can effectively balance the fusion quality between the defect block and the background region in complex backgrounds.

**Figure 12:**
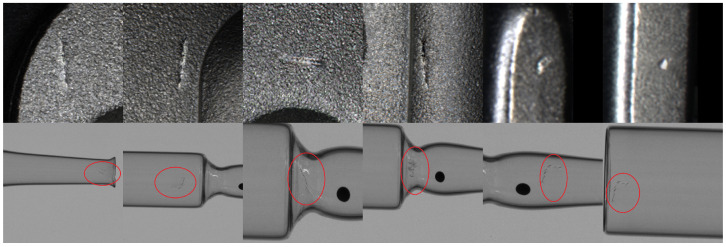
The results of integrating defect patches into normal samples using our method. The red circles in the figure denote the positions of the defects generated on the ampoule.

We also validated our method on a self-collected ampoule dataset, with results shown in Figure 12. The generated images demonstrate seamless defect integration and effective preservation of defect features.

To quantitatively evaluate the visual realism of the generated defects, an expert blind test was designed and conducted. Three senior engineers, each with over five years of experience in quality inspection on latch production lines, were invited as evaluators. The test material comprised 200 complete latch images with defects, of which 100 were from the real test set and the other 100 were randomly generated by our method. These images were randomly mixed. Under a single-blind condition, each evaluator was required to independently judge whether each image was “real” or “generated.” The average rate at which the generated samples were misclassified as real was used as the core evaluation metric. The results show that the average misclassification rate among the three experts was 64.3% (standard deviation ± 2.1%). This result, significantly higher than the random guessing level of 50%, strongly confirms from the perspective of human visual perception that our method is capable of generating defect samples with high authenticity.

### 3.5. Complete Defect Sample Replacement Experiments

To validate the authenticity of the generated samples and their effectiveness in replacement training, we designed and conducted a sample replacement experiment based on YOLO. This experiment utilized defect patch datasets of different categories for model training, and generated 312 complete defective samples through the second-stage defect patch fusion and optimization step. During the generation process, defects were precisely positioned and pasted onto target locations, with the system automatically recording the paste coordinates. Based on this location data, annotation files compliant with the YOLO format (normalized center coordinates + width/height) were generated programmatically, achieving fully automated end-to-end annotation. Subsequently, the real samples and generated samples were divided into training, validation, and test sets according to a ratio of 0.65, 0.15, and 0.2, respectively. A total of six experimental groups were established, as detailed in Table 8, with the Real-Real group serving as the baseline. The input image size for the YOLO model was uniformly set to 1024 × 1024. Common object detection data augmentation techniques (image translation, mirror flipping, color jittering) were applied during training. The experiment utilized mAP@50:95, mAP@50, precision, and recall as evaluation metrics. mAP@50 is a widely used evaluation metric in object detection tasks, measuring the detection performance when the Intersection over Union (IoU) threshold is set at 50%. A higher mAP@50 value indicates that the model can detect and localize targets more accurately. mAP@50:95 is a comprehensive evaluation metric that assesses model performance across multiple IoU thresholds. A higher mAP@50:95 value suggests that the model possesses a stronger capability for precise target localization.

(16) $$\mathrm{mAP}=\frac{1}{\left|T\right|}\sum_{t\in T}\mathrm{AP}\left(t\right)$$

Here, *T* is the set of IoU thresholds, and AP(t) represents the average precision at threshold *t*. As shown in Table 8, the comprehensive cross-domain evaluation reveals the multifaceted characteristics of the generated data in model training and its interplay with real data. The Real-Real group (mAP@50: 91.6, mAP@50:95: 50.4) establishes a definitive performance benchmark. Crucially, the Syn-Syn group (mAP@50: 91.4, mAP@50:95: 50.3) achieves nearly indistinguishable performance, demonstrating the exceptional internal consistency, feature fidelity, and self-domain effectiveness of the generated data. This validates its core utility in creating controlled, balanced, and scalable training environments.

The cross-domain evaluations further delineate the nature of the distributional shift. The Syn-Real group (mAP@50: 88.5) shows a measurable performance gap when transferring from synthetic to real domains, while the Real-Syn group (mAP@50: 90.1) exhibits a smaller gap in the reverse direction. This asymmetry confirms that while our generation process captures the macro-level defect distribution, subtle discrepancies in geometric fidelity, texture complexity, and background stochasticity between synthetic and real data remain. The model trained on the more complex real distribution generalizes better to the simpler synthetic domain than vice versa. The pronounced performance degradation in the Mixed-Real group (mAP@50: 80.2), where the model is trained on a blend of both domains but tested solely on real data, serves as a critical quantitative indicator. This result, consistent with findings in related work [61], demonstrates that a naive concatenation of synthetic and real data can be suboptimal. It suggests that without a sophisticated fusion mechanism, the combined dataset may introduce distributional bias and optimization conflict, preventing the model from effectively reconciling the two domains and potentially overfitting to the more regular patterns in the synthetic subset, thereby impairing its discriminative capability on pure real data.

These results provide a nuanced and actionable understanding: (1) The generated data is highly effective within its own domain and for tasks requiring domain-consistent data augmentation. (2) A measurable but manageable distribution gap exists, explaining the cross-domain generalization limits. (3) Most importantly, the performance drop in Mixed-Real is not merely a limitation but a clear diagnostic signal. It underscores that the key to unlocking the full potential of synthetic data lies not in simple mixing, but in developing advanced strategies such as curriculum learning, domain-invariant representation learning, or adaptive data weighting to intelligently bridge the domain gap. Thus, our work not only delivers a high-quality synthetic dataset but also empirically charts a clear path for future research in hybrid data utilization.

**Figure 13:**
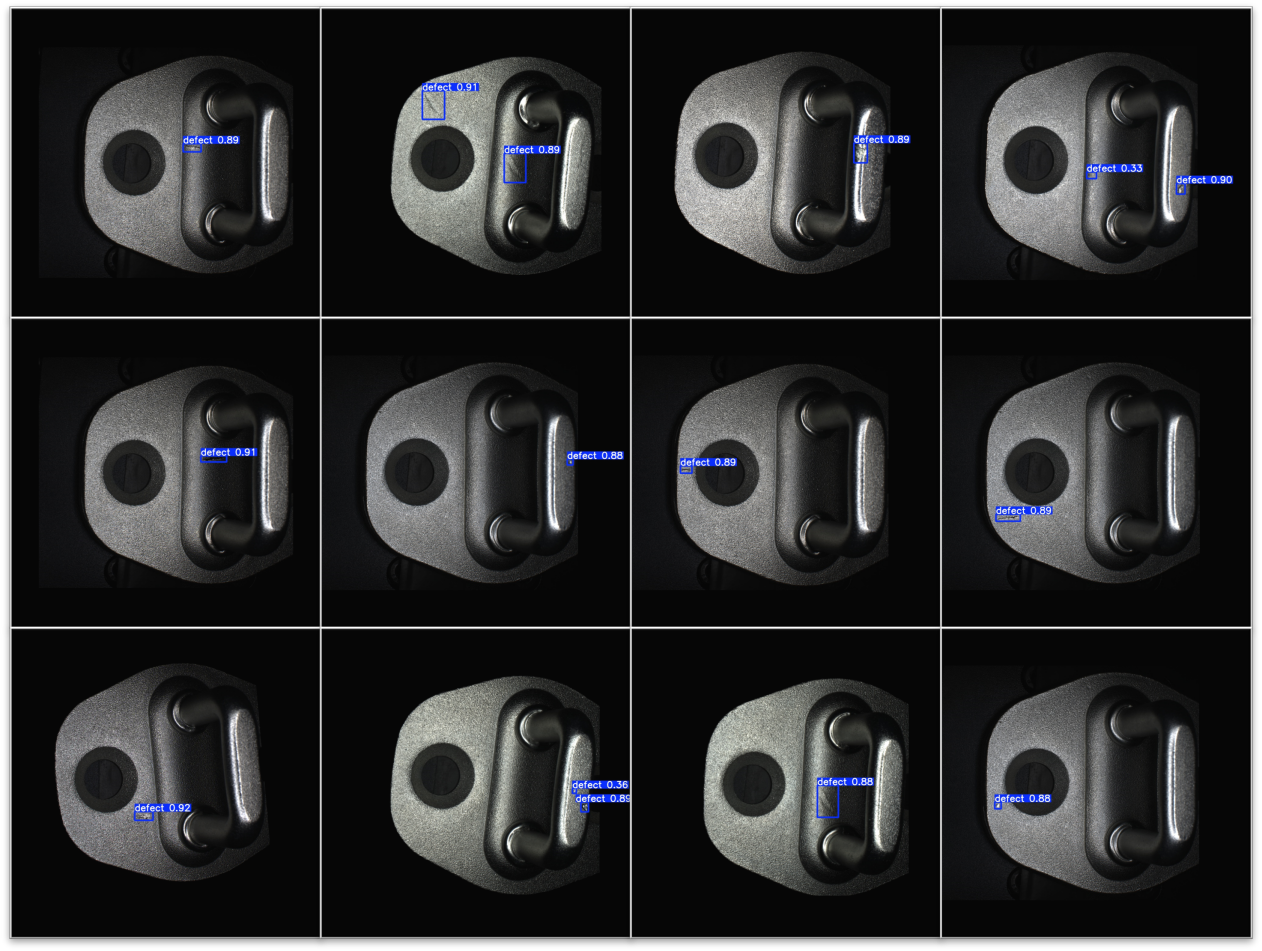
Object detection results. Training on real samples and testing on generated samples.

The object detection results are visualized in Figure 13.

### 3.6. Sample Augmentation Experiments Based on Object Detection Model

**Figure 14:**
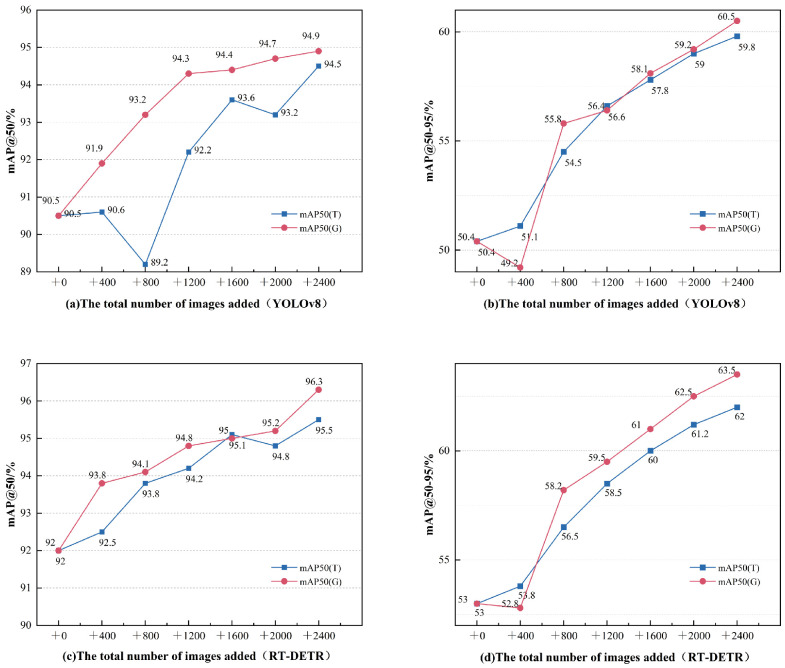
Performance comparison of data augmentation strategies on different object detection models. (**a**,**b**) present the mAP@50:95 and mAP@50 results of the YOLOv8 model, respectively. (**c**,**d**) illustrate the corresponding mAP@50:95 and mAP@50 results of the RT-DETR model. The curves demonstrate the effectiveness of our generative method (Ours) compared to traditional augmentation (Traditional) across varying sample sizes.

To evaluate the effectiveness of generated samples in augmentation training, we designed and conducted a sample augmentation experiment. The experiment utilized 312 real defect samples, of which 20% were randomly selected as a fixed test set. All experimental groups were evaluated using this identical test set. Different training sets were constructed by adding 400, 800, 1200, 1600, 2000, and 2400 generated samples, respectively, to the remaining real samples. To account for the data volume difference introduced by the generated samples, we also created equivalent-sized training sets using traditional data augmentation methods (random translation by 3 mm and random rotation) based on the real samples as a baseline group. These methods ensure minimal divergence between the augmented samples and the real images by introducing minor transformations. Within each training set group, 15% of the data was randomly selected as a validation set, and the input image size was set to 1024 × 1024 pixels. During training, mainstream object detection data augmentation techniques (such as image translation, mirror flipping, and color jittering) were applied. The experiment used mAP@50:95 and mAP@50 as the primary evaluation metrics. The YOLOv8 experimental results shown in Figure 14a,b indicate that both our generated sample method and the traditional augmentation method effectively improve model performance. When the sample size increased to 2400, the generative method outperformed the traditional method in both mAP@50:95 (60.5% vs. 59.8%) and mAP@50 (94.9% vs. 94.5%), demonstrating its performance advantage. Although the performance of both methods showed an overall upward trend with increasing sample size, the traditional method exhibited significant instability, with a notable performance drop at 800 samples (mAP@50 = 89.2%), likely due to distribution bias introduced by geometric transformations. In contrast, the generative method exhibited only slight fluctuations at small sample sizes (400) due to initial distribution bias, followed by stable improvement, indicating that the feature distribution of its generated samples is closer to the real data, providing a more robust augmentation effect. It is noteworthy that the generative method consistently achieved higher gains in mAP@50:95, reflecting its particular effectiveness in improving localization accuracy. When the sample size was large (2000–2400), the incremental improvements gradually diminished, suggesting that the performance might be approaching its upper limit under the current constraints. Overall, while maintaining stability, the generated samples demonstrated superior potential for performance improvement compared to traditional methods. To eliminate the dependency of our conclusions on a single model architecture, we conducted comparative experiments on the RT-DETR model, with results shown in Figure 14c,d. RT-DETR demonstrated a higher baseline performance (mAP@50 = 92.0%, mAP@50:95 = 53.0%), which aligns with expectations for an advanced detection model. Crucially, the core advantage of our method was perfectly replicated on RT-DETR. At the 2400 sample size, our method also achieved the highest performance on RT-DETR (mAP@50 = 96.3%, mAP@50:95 = 63.5%), and its advantage over the traditional method became more pronounced (the performance gap increased from 0.7% on YOLOv8 to 1.5%). This phenomenon suggests that RT-DETR’s powerful representational capacity can more fully utilize the rich information in high-quality generated samples, indicating a positive synergistic effect between our method and advanced model architectures. The experiments validate the effectiveness of the samples generated by our method in enhancing object detection performance. The data trends indicate that performance improvement is positively correlated with sample size, and the GAN shows greater potential in increasing dataset diversity. Although traditional augmentation is also effective, its growth is relatively limited. It is noteworthy that the impact of GAN-generated data has a complex relationship with sample size, with the most consistent performance improvements observed at medium to large sample sizes. Larger sample sizes may require further optimization of the generated samples to prevent performance instability caused by potential distribution bias.

### 3.7. Comparative Experiments

**Figure 15:**
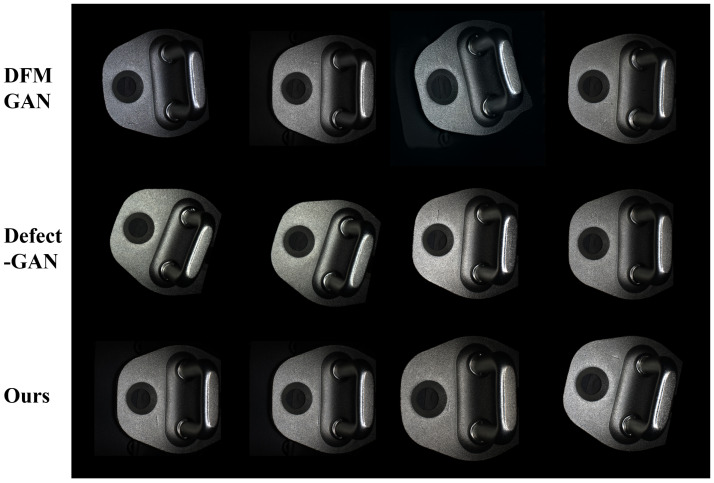
Comparison diagram of images generated by different methods. Compared to Defect-GAN and DFMGAN, our method produces more natural blending between defects and the background, better preserves structural integrity in complex regions, and avoids common artifacts such as blurred boundaries or unnatural color shifts. These visual results demonstrate the superior detail preservation and realism of our generated images.

Defect-GAN [25], as a single-stage defect generation method, requires training on a dataset containing non-defective images, defective images, and their corresponding defect masks. This method employs a composite layer structure based on inpainting and restoration processes to synthesize diverse and realistic defect samples. The Defect-aware Feature Manipulation Generative Adversarial Network (DFMGAN) [21] represents another two-stage approach. It first trains StyleGAN2 on defect-free images to generate high-fidelity background textures, and then employs defect-aware residual blocks to learn defect mask generation and local feature manipulation from limited defective samples. Compared to our two-stage defect generation method, Defect-GAN directly synthesizes complete defective images, requiring simultaneous modeling of both background and defect features. This joint optimization can lead to unnatural transition artifacts in complex backgrounds, and due to the lack of independent optimization of background and defect features, its capability in modeling complex defect patterns (e.g., local textures and geometric morphologies in automotive door lock strikes) is limited. Compared to our approach, DFMGAN requires pre-generating complete defect-free images. In practical industrial scenarios (e.g., door lock strike inspection), non-defective samples are abundant and highly consistent (fixed viewing angle, stable illumination), whereas defective samples are scarce and localized, having minimal global impact. Generating complete defect-free images introduces computational redundancy and unnecessary noise, thereby reducing generation efficiency and compromising output quality. To evaluate the performance differences, we designed a comparative experiment using 300 defect-free and 120 defective images of automotive door lock strikes. The defect regions were annotated using LabelMe. Data augmentation techniques (such as image translation, mirror flipping, and color jittering) were also employed. Three datasets were prepared: a defect patch dataset (for our method), a dataset conforming to the MVTec standard (used by DFMGAN), and a dataset in Defect-GAN format. All experiments were conducted on an RTX 3090 GPU (with 24GB VRAM) (NVIDIA Corporation, Santa Clara, CA, USA) using PyTorch 1.9.0, Python 3.8, and CUDA 11.1. Image quality was quantified using the Fréchet Inception Distance (FID), and computational resource requirements were additionally analyzed. In the task of generating defects for automotive door lock strikes, our method demonstrated significant performance advantages (Table 9). Compared to DFMGAN, our two-stage generation strategy reduced the FID score by 18.3% (31.51 vs. 38.59), while also significantly improving training efficiency. As shown in Figure 15, although the defect details generated by DFMGAN exhibit high realism, insufficient training in its first-stage background generation leads to two key issues: (1) distortion of background shape and texture, and (2) unnatural color shifts (an anomalous bluish tint). The single-stage architecture of Defect-GAN has fundamental limitations in modeling complex geometries, resulting in poor training efficiency metrics (FID = 59.18, training time 10 h). Figure 15 shows that images generated by Defect-GAN often exhibit missing defects, primarily due to insufficient training samples and inadequate training duration under the experimental conditions. Our method focuses on learning local defect features (such as texture and shape) to enhance computational efficiency and optimize resource utilization. By directly generating defect image patches without modeling the global background, our method significantly reduces the computational burden. This method achieves a balance between generated image quality and computational resource utilization, making it particularly suitable for resource-constrained industrial environments.

### 3.8. Summary of Experimental Results

The experiments in Section 3.1 detail the construction of the dataset, system calibration, and experimental configuration, laying the foundation for all subsequent work. The experiments in Section 3.2 conducted a comprehensive evaluation of the imaging scheme through qualitative and quantitative experiments, verifying the effectiveness of the imaging system and providing 2D data support for subsequent work. The experiments in Section 3.3 validated the effectiveness of the first-stage method in generating high-quality defect patches. Quantitative metrics (FID, LPIPS, IS) and visual assessment demonstrated the realism and diversity of the generated samples. In the first stage, compared to the original FastGAN, our method improved the FID score by 11–24% across different defect categories, indicating that the generated defect patches are more similar to real ones in texture and structural details, which is critical for industrial imaging applications. For instance, the FID for linear defects decreased from 55.85 to 46.25, indicating that the generated defects more closely resemble the appearance of real defects under varying imaging conditions. Furthermore, the LPIPS and IS scores further confirmed the improvement in perceptual quality and diversity, ensuring that the generated patches can capture a variety of defect manifestations. We also conducted ablation studies to validate the learning mechanism of the alpha and beta parameters, demonstrating their dynamic adaptation during training. On other datasets (such as AFHQ_v2 and the automotive door lock strike dataset), FID showed consistent improvements of 5% to 20%, highlighting the generalization ability of the method across different imaging scenarios. In the second stage, the experiments in Section 3.4 showed that our method significantly improved the FID compared to other methods, ensuring that the generated defect patches maintain realistic visual characteristics after being fused with normal samples. In blind tests, experienced quality inspectors misclassified 64.3% (±2.1%) of the generated samples as real ones, providing objective evidence for the high quality of the images produced by our method. The experiments in Section 3.5 further highlighted the practical impact of our method on deep learning-based defect detection models. By introducing varying amounts of generated samples into the training of object detection models, we observed a significant improvement in detection performance compared to using only real samples. Specifically, on YOLOv8, the mAP@50:95 metric saw a maximum increase of 10.1 percentage points (rising from 50.4% to 60.5%), and mAP@50 also showed significant improvement when training with a mix of real and generated samples. These improvements indicate that the generated samples effectively augment the training dataset, enhancing the model’s ability to detect defects in challenging imaging scenarios, such as non-planar metal workpieces with reflective surfaces or complex geometries. It is noteworthy that the training process remained efficient, requiring only a few hours on an RTX 3080 GPU to generate high-quality defect samples, making it practical for industrial applications requiring rapid deployment. The experiments in Section 3.6 and Section 3.7 further validated the framework. Section 3.6 demonstrated the effectiveness of the generated samples in augmenting the training data for object detection models. Section 3.7 included comparative experiments with other defect generation methods (DFMGAN and Defect-GAN), demonstrating the performance superiority of our method in generating defects for automotive door lock strikes.

## 4. Conclusions and Future Work

This research overcomes the systemic bottleneck of fragmentation across imaging, generation, and recognition stages by introducing a task-driven, end-to-end paradigm for defect detection on highly reflective, complex metal workpieces. Through the co-design and closed-loop optimization of imaging physics, data synthesis, and recognition models, the solution transforms the challenging 3D defect recognition problem into a robust, deployable 2D vision system, significantly enhancing feasibility under industrial constraints of limited samples and harsh imaging conditions. The main contributions of this work, which are closely interlinked, are summarized as follows:

(1) A 3D-to-2D Defect Mapping Strategy via Systemic Co-design: The core of this work is a systematic modeling philosophy. It achieves a stable and reliable mapping from 3D surface defects to discriminable 2D image features through the co-optimization of the imaging subsystem, generative algorithms, and methods adapted for highly reflective metals. This strategy successfully converts a spatially complex 3D inspection task into a more tractable 2D recognition problem without reliance on 3D sensors or CAD models. (2) A Robust Imaging Subsystem for Complex Reflective Workpieces: We developed a dedicated imaging device integrating precision fixturing, dome diffuse lighting, and a small-aperture lens. This hardware subsystem effectively suppresses specular highlights and irregular shadows at the source, providing uniformly illuminated, high-contrast images that serve as “clean” input for subsequent data generation and detection, thereby laying the physical foundation for system robustness. (3) A Task-Driven Software Architecture for Defect Imaging and Generation: Constructed with the ultimate goal of “training a few-shot detection model,” this architecture integrates a two-stage generation process: a defect-patch synthesis stage based on an improved FastGAN with a Dynamic Multi-Granularity Fusion module, which efficiently learns defect representations; and a realistic fusion stage employing optimized Poisson editing and composite loss for precise, natural-looking defect injection. This integrated pipeline provides a clear, systematic engineering pathway for few-shot defect data synthesis.

Both quantitative and qualitative experiments demonstrate that our integrated system surpasses traditional fragmented solutions in imaging quality, data generation fidelity (e.g., FID score), and ultimately, the performance of downstream few-shot detectors. From an industrial application perspective, this work significantly reduces dependency on massive manual annotation, decreases the cost of model training, and improves deployment practicality.

Although this study has made significant progress in improving defect sample generation quality and system integration, it still faces some challenges. For example, when dealing with specific categories classified as “compound defects,” due to their large feature variation and complex morphology, the model training is not stable enough, and generating high-fidelity pixel-level details remains difficult, which limits the accurate representation of complex defect patterns.

Future research work will focus on the following aspects: Firstly, we will further optimize the structure of the feature extraction and generation modules to enhance the model’s ability to adapt to and reconstruct diverse defect features, thereby improving the generation quality for complex defects. Secondly, we are investigating a robotic arm-assisted “pose unification strategy,” where a robotic grasping and positioning system stably places the workpiece in the same location and orientation, effectively reducing imaging interference caused by pose variations and improving data consistency and generation stability. Furthermore, we will also dedicate efforts to optimizing the multi-light source configuration and illumination parameters in the optical imaging system to further enhance the ability to extract faint defects and improve feature visibility. We plan to extend this method to more types of non-planar metal workpieces, such as door hinges, support arms, and connectors, which have complex 3D structures, and conduct extensive validation under diverse fixturing and lighting conditions to further test the system’s generalization ability and robustness. We will also explore controllable defect parameter generation technology, enabling it to adapt to different manufacturing standards and quality assessment needs, further expanding its application potential in industrial visual inspection.

## Figures and Tables

**Table 1:** Evolution of α parameters during training across resolutions.

Iteration	$\alpha_{64}$	$\alpha_{128}$	$\alpha_{256}$	$\alpha_{512}$
0	0.5000	0.5000	0.5000	0.5000
10,000	0.6550	0.5270	0.5640	0.5030
50,000	0.7690	0.6270	0.6570	0.4940
100,000	0.8172	0.7064	0.6821	0.4910

**Table 2:** Defect statistics and training parameters.

Defect Type (Numbers)	Size Range	Avg. Diam.	Training Config
	(Min–Max)	(Mean ± Std)	(Input Size, Batch)
Linear (216)	0.73 × 0.57–6.19 × 3.31	3.14 ± 0.98	512^2^, 8
Compound (234)	1.02 × 0.65–6.83 × 3.62	2.87 ± 1.21	256^2^, 16
Punctate (116)	0.73 × 0.58–2.88 × 1.93	1.52 ± 0.47	128^2^, 32

Note: All dimensions are in millimeters. Size range shows minimum and maximum dimensions (length × width). Training configuration shows input size (pixels) and batch size. The minimum detectable defect size (0.73 × 0.57 mm) is below the industrial threshold of 1.0 mm.

**Table 3:** Effect of aperture selection on depth-of-field, image quality, and defect detection performance under identical dome illumination.

Configuration	DOF [mm]	$d_{\mathrm{Airy}}$ [μm]	Sharpness/Noise	mAP@50 [%]
f/11	45.7	14.76	High sharpness, low noise	88.3 ± 0.62
f/16	66.5	21.47	Balanced	91.6 ± 0.71
f/22	91.6	29.52	Low sharpness, high noise	89.2 ± 0.67

**Table 4:** Comparison of Imaging Quality and Detection Performance Across Different Optical Configurations.

Configuration	Sharpness	Noise	mAP@50	mAP@50:95	F1	FP Rate	Image $\sigma_{\mathrm{bg}}$
Dome+f/16	266.40 ± 5.8	2.50 ± 0.12	91.6 ± 0.7	50.4 ± 1.1	0.89 ± 0.02	0.7 ± 0.15	0.0477
Ring+f/16	303.70 ± 8.2	3.71 ± 0.18	86.7 ± 0.9	47.1 ± 1.3	0.85 ± 0.03	2.3 ± 0.28	0.0545
Bar+f/16	176.55 ± 7.3	3.20 ± 0.16	89.3 ± 0.8	35.2 ± 1.8	0.76 ± 0.03	3.0 ± 0.35	0.0588
Dome+f/4	131.68 ± 6.5	2.74 ± 0.13	88.5 ± 0.8	47.7 ± 1.2	0.81 ± 0.02	1.5 ± 0.22	0.0496

Note: The Sharpness, Noise, mAP, F1, and FP Rate are reported as mean ± standard deviation from 5 independent experimental runs. The Image $\sigma_{\mathrm{bg}}$ column reports the background noise level ($\sigma_{\mathrm{bg}}$) computed from representative individual images under corresponding conditions, illustrating per-image variability.

**Table 5:** Ablation Study Results on FID, LPIPS, and IS Metrics Across Different Defect Types. Lower FID and LPIPS indicate more realistic generated images; higher IS indicates greater diversity.

Method	Linear Defects			Punctate Defects			Compound Defects		
	FID	LPIPS	IS	FID	LPIPS	IS	FID	LPIPS	IS
Fast (Baseline)	55.85 (±3.73)	0.2043 (±0.0136)	2.382 (±0.235)	50.99 (±3.41)	0.2074 (±0.0139)	2.202 (±0.108)	75.27 (±5.03)	0.2068 (±0.0138)	2.333 (±0.119)
Fast + DMGF	51.98 (±2.93)	0.1946 (±0.0110)	2.507 (±0.204)	49.43 (±2.78)	0.1975 (±0.0111)	2.318 (±0.094)	58.70 (±3.30)	0.1964 (±0.0111)	2.313 (±0.104)
Fast + PER	47.19 (±2.80)	0.1946 (±0.0115)	2.367 (±0.158)	46.27 (±2.74)	0.1975 (±0.0117)	2.332 (±0.131)	58.47 (±3.47)	0.1969 (±0.0117)	2.221 (±0.121)
Fast + All	46.25 (±1.83)	0.1911 (±0.0076)	2.538 (±0.126)	44.97 (±1.78)	0.1922 (±0.0076)	2.557 (±0.087)	56.49 (±2.24)	0.1915 (±0.0076)	2.463 (±0.086)

Note: All values are presented as mean (±standard deviation). Lower FID and LPIPS values indicate better performance, while higher IS values are preferred. The standard deviations for FID and LPIPS were calculated based on the coefficient of variation from the IS metric.

**Table 6:** Our method vs. FastGAN: FID comparison across different datasets.

Dataset	Numbers	Size	FID (Fast/Ours)
Car Door Latches	180	$1024^{2}$	46.19 ± 1.2/34.26 ± 0.8
AFHQ_v2 (Dog)	389	$256^{2}$	54.32 ± 1.5/49.80 ± 1.1
AFHQ_v2 (Dog)	389	$512^{2}$	55.72 ± 1.6/52.56 ± 1.2
AFHQ_v2 (Cat)	493	$256^{2}$	26.35 ± 0.9/23.85 ± 0.7
AFHQ_v2 (Cat)	493	$512^{2}$	31.75 ± 1.1/28.35 ± 0.8

**Table 7:** Quality Test FID Results on Car Door Latch Positions.

Method	TOP	Middle	Bottom
CutPaste	87.06 ± 2.5	108.06 ± 3.2	160.98 ± 5.1
Poisson	28.32 ± 1.0	34.78 ± 1.2	60.57 ± 1.8
Ours	25.34 ± 0.8	33.63 ± 0.9	54.73 ± 1.5

**Table 8:** Group settings for the data augmentation sample replacement experiment and experiment results.

Group	Train		Val		Test		Results	
	Real	Syn	Real	Syn	Real	Syn	mAP@50	mAP@50:95
Real-Real	✓		✓		✓		91.6 ± 0.7	50.4 ± 1.1
Syn-Syn		✓		✓		✓	91.4 ± 0.8	50.3 ± 1.2
Real-Syn	✓		✓			✓	90.1 ± 0.9	49.6 ± 1.3
Syn-Real		✓		✓	✓		88.5 ± 1.0	48.7 ± 1.4
Mixed-Real	✓	✓	✓	✓	✓		80.2 ± 1.5	44.6 ± 2.0
Mixed-Syn	✓	✓	✓	✓		✓	84.7 ± 1.3	46.5 ± 1.8

**Table 9:** Comparison of different methods.

Method	Normal Samples	Defective Samples	Training Duration	FID
Ours	300	120	Stage 1: 4 h 37 m	31.5072 ± 3.3240
			Stage 2: 0	
DFMGAN	300	120	Stage 1: 21 h 23 m	38.5939 ± 4.2702
			Stage 2: 9 h 20 m	
Defect-GAN	864	120	10 h 27 m	59.1835 ± 7.0526

## Data Availability

The raw data supporting the conclusions of this article will be made available by the authors upon request.
